# Regulation of the transcription factor CdnL promotes adaptation to nutrient stress in *Caulobacter*

**DOI:** 10.1093/pnasnexus/pgae154

**Published:** 2024-04-10

**Authors:** Erika L Smith, Gaël Panis, Selamawit Abi Woldemeskel, Patrick H Viollier, Peter Chien, Erin D Goley

**Affiliations:** Department of Biological Chemistry, Johns Hopkins University School of Medicine, Baltimore, MD 21205, USA; Department of Microbiology and Molecular Medicine, University of Geneva, Geneva 1211, Switzerland; Department of Biological Chemistry, Johns Hopkins University School of Medicine, Baltimore, MD 21205, USA; Department of Microbiology and Molecular Medicine, University of Geneva, Geneva 1211, Switzerland; Department of Biochemistry and Molecular Biology, University of Massachusetts-Amherst, Amherst, MA 01003, USA; Department of Biological Chemistry, Johns Hopkins University School of Medicine, Baltimore, MD 21205, USA

**Keywords:** CarD, CdnL, stringent response, proteolysis, adaptation

## Abstract

In response to nutrient deprivation, bacteria activate a conserved stress response pathway called the stringent response (SR). During SR activation in *Caulobacter crescentus*, SpoT synthesizes the secondary messengers guanosine 5′-diphosphate 3′-diphosphate and guanosine 5′-triphosphate 3′-diphosphate (collectively known as (p)ppGpp), which affect transcription by binding RNA polymerase (RNAP) to down-regulate anabolic genes. (p)ppGpp also impacts the expression of anabolic genes by controlling the levels and activities of their transcriptional regulators. In *Caulobacter*, a major regulator of anabolic genes is the transcription factor CdnL. If and how CdnL is controlled during the SR and why that might be functionally important are unclear. In this study, we show that CdnL is down-regulated posttranslationally during starvation in a manner dependent on SpoT and the ClpXP protease. Artificial stabilization of CdnL during starvation causes misregulation of ribosomal and metabolic genes. Functionally, we demonstrate that the combined action of SR transcriptional regulators and CdnL clearance allows for rapid adaptation to nutrient repletion. Moreover, cells that are unable to clear CdnL during starvation are outcompeted by wild-type cells when subjected to nutrient fluctuations. We hypothesize that clearance of CdnL during the SR, in conjunction with direct binding of (p)ppGpp and DksA to RNAP, is critical for altering the transcriptome in order to permit cell survival during nutrient stress.

**Significance Statement**

The stringent response (SR) is a ubiquitous bacterial stress response that promotes adaptation to nutrient deprivation. While it is known that SR activation affects RNA polymerase activity to reprogram the transcriptome, the impact of the SR on other transcriptional regulators is not well understood. In this study, we show that a conserved transcription factor, CdnL, is cleared upon activation of the SR, and its clearance is important for cells to efficiently adapt to nutrient fluctuations. Our results suggest that CdnL regulation enables adaptation by transcriptionally down-regulating ribosome biosynthesis and flux through metabolic pathways, thereby promoting survival during nutrient stress. As CdnL homologs are broadly found, we hypothesize that CdnL regulation is a conserved mechanism of bacterial adaptation to stress.

## Introduction

Bacterial replication requires available nutrients and the transcription of anabolic genes. When nutrients become limited, cells adapt to their environment by activating the broadly conserved bacterial stress response pathway known as the stringent response (SR) (1). During the SR, stressors, such as nutrient limitation and heat shock, are sensed by Rel/Spo homolog (RSH) proteins, which synthesize and/or hydrolyze the second messengers guanosine 5′-diphosphate 3′-diphosphate and guanosine 5′-triphosphate 3′-diphosphate (collectively known as (p)ppGpp) (1, 2). (p)ppGpp broadly impacts DNA replication, transcription, translation, and metabolism by directly or indirectly interacting with various proteins to halt growth until conditions improve (1).

*Caulobacter crescentus* (hereafter *Caulobacter*) is a gram-negative freshwater α-proteobacterium with a dimorphic life cycle that allows it to exist as a nutrient-seeking swarmer cell or as a reproductive stalked cell (1). Nutrient limitation and (p)ppGpp accumulation slow the swarmer-to-stalked transition, likely to allow the swarmer cell time to seek out nutrients before differentiating (1–4). This transition is governed by regulatory proteins, whose levels and activities are impacted by (p)ppGpp upon SR activation (3, 5). The availability of nutrients thus regulates the *Caulobacter* cell cycle, promoting anabolic processes and cell cycle progression in nutrient-rich environments, while favoring catabolic processes and nutrient-seeking behavior when resources are limited.

*Caulobacter* inhabits oligotrophic environments and is regularly exposed to fluctuations in nutrient availability; therefore, cells must balance anabolism with SR activation by readily responding to nutrient deprivation (1, 6). In *Caulobacter*, nutrient limitation is sensed by a single bifunctional RSH protein that is known as SpoT, which synthesizes (p)ppGpp during nutrient stress to initiate the SR (1, 5, 6). Ultimately, (p)ppGpp accumulation causes down-regulation of genes required for translation, growth, and division, and up-regulation of genes important for responding to stress, thus impacting the activities of proteins involved in both anabolism and SR activation (1, 7–9). A major way that (p)ppGpp exerts these effects on gene regulation is by binding directly to two sites on RNA polymerase (RNAP). These are referred to as “site 1” and “site 2” in *Escherichia coli*, and both sites are conserved in *Caulobacter* (1, 5–11). Site 2 is formed at the interface between RNAP and the transcription factor DksA, and the binding of (p)ppGpp to this site specifically has been implicated in enhancing the influence of DksA on RNAP and leading to the inhibition of rRNA promoter activity and decreased anabolism (1, 7, 9, 12).

(p)ppGpp can also cause a down-regulation of anabolic gene transcription by directly or indirectly controlling the levels and activities of other transcriptional regulators (10). In *Caulobacter*, a major regulator of anabolic gene transcription and cell cycle progression is the broadly conserved transcription factor CdnL, which stands for “CarD N-terminal like” as it shares homology with the N-terminal domain of the *Myxococcus xanthus* transcription factor, CarD (13–16). Under nutrient-rich conditions, CdnL directly binds to RNAP and promotes transcription of housekeeping genes and growth (13, 17). Similarly, the *Mycobacterium tuberculosis* CdnL homolog CarD was shown to be an activator of rRNA genes whose levels are positively correlated with mycobacterial growth (17–21). We have previously shown that loss of *Caulobacter* CdnL (Δ*cdnL*) results in slowed growth and altered morphology, as well as down-regulation of anabolic genes, such as those involved in macromolecule biosynthesis and ribosome biogenesis (14, 16). These data are further supported by metabolomic analyses showing altered metabolite levels in Δ*cdnL* cells (14).

The physiological changes observed in Δ*cdnL* cells mirror those of cells undergoing SR activation. Indeed, mycobacterial CarD was recently found to be down-regulated during stress conditions, although a direct link to the SR was not examined (22). These putative connections made us consider whether CdnL is regulated in a similar manner during the *Caulobacter* SR. In this study, we demonstrate that CdnL is regulated posttranslationally during the SR and this regulation promotes effective adaptation to changes in nutrient availability by altering anabolic gene transcription.

## Results

### CdnL is cleared during the SR in a SpoT-dependent manner

**Fig. 1.**
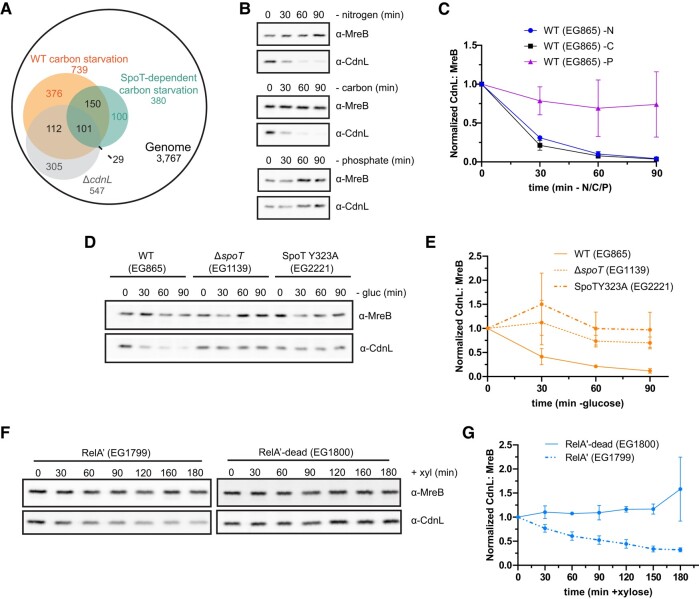
CdnL is cleared during conditions that activate the SR. A) Venn diagram of genes down-regulated in Δ*cdnL*, in WT carbon-starved cells, and in a SpoT-dependent manner (6, 14, 23). Two hundred and thirteen genes overlapped between the Δ*cdnL* dataset and the WT carbon-starved dataset (14, 23). One hundred and thirty genes overlapped between the Δ*cdnL* dataset and the SpoT-dependent carbon-starved dataset (6, 14). Both are significant enrichments (*P* < 0.05) as determined by hypergeometric probability. B) Representative western blots of CdnL during 90 min of nitrogen, carbon, and phosphate starvation. MreB was used as a loading control. C) Densitometry of CdnL levels (normalized to MreB) relative to *t* = 0 during nitrogen (–N), carbon (–C), and phosphate (–P) starvations from western blots as performed in (B). Error bars represent ±1 SD of three biological replicates. D) Representative western blot of CdnL during 90 min of glucose (gluc) starvation in a WT, Δ*spoT*, or SpoTY323A background. MreB was used as a loading control. E) Densitometry of CdnL levels (normalized to MreB) relative to *t* = 0 from western blots as performed in (D). Error bars represent ±1 SD of three biological replicates. F) Representative western blot of CdnL during 180 min of xylose (+xyl) induction of RelA′ or RelA′-dead. MreB was used as a loading control. G) Densitometry of CdnL levels (normalized to MreB) relative to *t* = 0 from western blots as performed in (F). Error bars represent ±1 SD of three biological replicates.

Our prior work indicated that many anabolic genes are regulated in a CdnL-dependent manner, apparently echoing transcriptional changes that occur during the SR. This prompted us to review transcriptomic data from our laboratory and others to explore a potential relationship between CdnL and SR. In comparing Δ*cdnL* cells to wild-type (WT) starved cells, we find 213 genes down-regulated during carbon starvation are also down-regulated in Δ*cdnL*, which is a significant enrichment as determined by hypergeometric probability (Fig. 1A) (14, 23). This enrichment is, at least in part, SpoT-dependent, as 130 genes that are down-regulated in Δ*cdnL* cells are down-regulated during carbon starvation in a SpoT-dependent manner, which is also statistically significant (Fig. 1A) (6, 14). These overlaps suggest a link between SR activation by SpoT and the absence of CdnL.

To investigate a potential connection between SR activation and the loss of CdnL, we assessed CdnL protein levels during nitrogen, carbon, and phosphate starvation. Both carbon and nitrogen limitation activate the SR in *Caulobacter*, while phosphate limitation does not (1). To do this, *Caulobacter* cells were grown in minimal media, then washed and incubated in media lacking a carbon, nitrogen, or phosphate source. Cell lysates were sampled at up to 90 min of starvation, and CdnL levels were assessed by immunoblotting. We found that CdnL was cleared in WT cells under both nitrogen and carbon starvation with half-lives of 18 and 14 min, respectively (Fig. 1B and C; SI Appendix, Table S1). Notably, CdnL levels remained stable under phosphate starvation (Fig. 1B and C; SI Appendix, Table S1).

The fact that CdnL is cleared upon carbon and nitrogen starvation, but not phosphate starvation, is consistent with CdnL regulation being downstream of SR activation. Therefore, we asked whether CdnL clearance depended on the only *Caulobacter* RSH enzyme, SpoT, by repeating these starvation experiments in cells lacking SpoT (Δ*spoT*) (6). During nitrogen and carbon starvation, CdnL levels were significantly more stable in Δ*spoT* than in WT, with the half-lives increasing to 60 min and over 500 min, respectively (Fig. 1D and E; SI Appendix, Fig. S1A, B and Table S1). CdnL was also stabilized during carbon starvation in the presence of a SpoT variant that is unable to synthesize (p)ppGpp (SpoTY323A) (Fig. 1D and E; SI Appendix, Table S1) (2). These observations indicate that both SpoT and (p)ppGpp are necessary for CdnL clearance during starvation.

We next tested whether (p)ppGpp is sufficient for CdnL clearance using a strain harboring a xylose-inducible, constitutively active version of the *E. coli* (p)ppGpp synthetase RelA (RelA′) or a catalytically inactive RelA variant (RelA′-dead) as a negative control (4). We found that CdnL was cleared under nutrient-replete conditions upon expression of RelA′ with a half-life of 103 min, while CdnL was stable in the presence of the catalytically inactive variant, indicating that (p)ppGpp is sufficient to clear CdnL (Fig. 1F and G; SI Appendix, Table S1). Taken together, these results suggest that CdnL is cleared during conditions that activate the SR, and (p)ppGpp is both necessary and sufficient to induce clearance. For simplicity, we chose to use carbon starvation to probe the mechanism and importance of CdnL clearance during stringent activation.

### Transcriptional control of *cdnL* is not sufficient to regulate CdnL levels during the SR

We observed that CdnL protein levels decrease upon carbon starvation, but it was unclear whether the regulation of CdnL during starvation occurs transcriptionally, posttranscriptionally, and/or posttranslationally. Indeed, previous studies showed that *cdnL* transcript levels also decreased upon carbon starvation (23). We first asked whether transcriptional activity at the *cdnL* promoter contributes significantly to controlling CdnL levels during the SR. To do this, we expressed *cdnL* from a xylose-inducible promoter (P*_xyl_ -cdnL*) in Δ*cdnL*, Δ*spoT*Δ*cdnL*, and SpoTY323A Δ*cdnL* backgrounds. In this system, we can shut off *cdnL* transcription independently of SR activation or (p)ppGpp production by washing out xylose and resuspending cells in nutrient-replete media lacking xylose (M2G), or in carbon starvation media lacking both xylose and glucose (M2). Under carbon starvation conditions and after the removal of xylose to prevent further *cdnL* transcription, CdnL was rapidly cleared in Δ*cdnL* P*_xyl_ -cdnL* cells with a half-life of 13 min. In contrast, for Δ*spoT*Δ*cdnL* P*_xyl_ -cdnL* and SpoTY323A Δ*cdnL* P*_xyl_ -cdnL* cells, CdnL levels remained stable with half-lives of 139 and 334 min, respectively (SI Appendix, Fig. S2A, B and Table S1). This suggests that transcriptional control is not sufficient for CdnL clearance. However, there appear to be additional factors governing CdnL stability during the SR. While CdnL was cleared more rapidly in starvation conditions compared with nutrient-replete conditions in Δ*cdnL* P*_xyl_ -cdnL* cells after the removal of xylose (13 min compared with 27 min), we found that in Δ*spoT*Δ*cdnL* P*_xyl_ -cdnL* and SpoTY323A Δ*cdnL* P*_xyl_ -cdnL* cells, CdnL was actually cleared more rapidly in nutrient-replete conditions compared with starvation conditions after the removal of xylose (70 and 75 min compared with 139 and 334 min, respectively; SI Appendix, Fig. S2A–D and Table S1). These data imply that CdnL levels are regulated posttranscriptionally in a manner that is partially SpoT dependent.

### CdnL is a ClpXP target during starvation

**Fig. 2.**
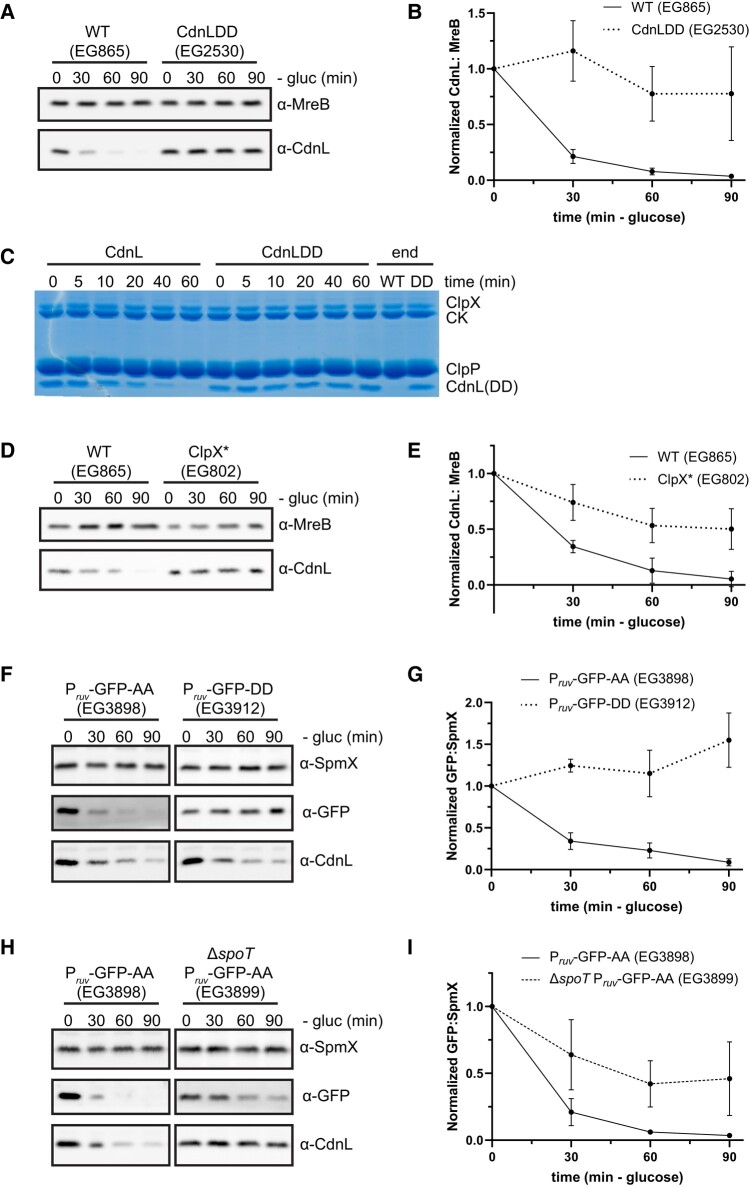
ClpXP is implicated in CdnL clearance. A) Representative western blot of CdnL or CdnLDD during 90 min of glucose (gluc) starvation. MreB was used as a loading control. B) Densitometry of CdnL or CdnLDD levels (normalized to MreB) relative to *t* = 0 from western blots as performed in (B). Error bars represent ±1 SD of three biological replicates. C) In vitro degradation of CdnL or CdnLDD. CK indicated creatine kinase, which is used for ATP regeneration in the reaction. Experiments were performed twice each. D) Representative western blot of CdnL during 90 min of glucose (gluc) starvation following a 2-h induction of ClpX* with 0.3% xylose. MreB was used as a loading control. E) Densitometry of CdnL levels (normalized to MreB) relative to *t* = 0 from western blots as performed in (D). Error bars represent ±1 SD of three biological replicates. F) Representative western blot of GFP-AA or GFP-DD during 90 min of glucose (gluc) starvation. SpmX was used as a loading control. CdnL was used as a comparison. G) Densitometry of GFP-AA or GFP-DD levels (normalized to SpmX) relative to *t* = 0 from western blots as performed in (F). Error bars represent ±1 SD of three biological replicates. H) Representative western blot of GFP-AA in a WT or Δ*spoT* background during 90 min of glucose (gluc) starvation. SpmX was used as a loading control. CdnL was used as a comparison. I) Densitometry of GFP-AA levels (normalized to SpmX) relative to *t* = 0 from western blots as performed in (H). Error bars represent ±1 SD of three biological replicates.

Since transcriptional regulation is not sufficient to control CdnL levels during the SR, we next asked whether CdnL is regulated posttranslationally. CdnL bears two alanine residues on the C-terminus, which is a common degradation signal (degron) recognized by the ClpXP protease (24, 25). It was previously shown that the CdnL C-terminus was important for ClpXP-mediated degradation of CdnL in vivo (13). However, it remains unclear under what circumstances CdnL proteolysis is functionally relevant, which caused us to wonder whether CdnL degradation by ClpXP occurs during starvation. We, therefore, asked whether eliminating the putative C-terminal degron impacts CdnL levels during starvation by changing the two alanine residues to aspartate residues, thus creating the variant CdnLDD (25). We found that CdnLDD was not cleared during either nitrogen or carbon starvation, suggesting that ClpXP targets CdnL for the degradation during the SR (Fig. 2A and B; SI Appendix, Fig. S1A and C). We asked whether CdnL is a direct proteolytic target of ClpXP in vitro and found that purified ClpXP was capable of degrading purified CdnL, but not purified CdnLDD, in an ATP-dependent manner (Fig. 2C; SI Appendix, Fig. S3A). The ability of ClpXP to degrade CdnL in vitro is not affected by (p)ppGpp, as the addition of (p)ppGpp did not further stimulate CdnL turnover (SI Appendix, Fig. S3B). Finally, we assessed whether ClpX activity was required in vivo for CdnL clearance during carbon starvation using a dominant-negative ATPase-dead ClpX variant (ClpX*) (26, 27). When cells were starved of carbon after a 1-h induction of ClpX*, CdnL levels were again stabilized (Fig. 2D and E). These data confirm that CdnL is a ClpXP target and establish that ClpXP-mediated proteolysis is required for CdnL regulation during carbon starvation.

It is evident that the CdnL C-terminus is critical for the regulation of CdnL by ClpXP. To assess whether the CdnL C-terminus is sufficient for ClpXP-mediated degradation during starvation, we tagged GFP with the last 15 amino acids from the CdnL tail (GFP-AA) or with the last 15 amino acids from the stabilized CdnL tail (GFP-DD). We then expressed these constructs on a plasmid under control of the *ruv* operon promoter, as transcript levels for the *ruvA* DNA helicase gene are not affected during carbon starvation (6). Strikingly, GFP-AA was rapidly degraded during carbon starvation while GFP-DD remained stable, demonstrating that the CdnL tail is indeed sufficient for degradation of GFP (Fig. 2F and G). We next sought to test the SpoT-dependency of this degradation. To do this, we put the GFP-AA construct into the Δ*spoT* background and repeated the carbon starvation. GFP-AA was degraded more slowly in a Δ*spoT* background (68 min half-life) than in a WT background (14 min half-life), indicating that there is at least a partial SpoT dependency to ClpXP-mediated degradation (Fig. 2H and I; SI Appendix, Table S1).

### The CdnL–RNAP interaction is not sufficient to protect CdnL from proteolysis

There appear to be other factors governing CdnL stability outside of the C-terminus since we found that GFP-AA was cleared, albeit slowly, in Δ*spoT* cells, while CdnL itself remained stable (Figs. 1D and E and 2H and I). Since CdnL interacts with RNAP while GFP does not, we hypothesized that the interaction with RNAP may reduce CdnL degradation, possibly by protecting CdnL from proteolysis by ClpXP. To assess this, we constructed two CdnL point mutants, V39A and P54A, which have a reduced affinity for RNAP (13). We then assessed their stability during carbon starvation. Both the V39A and P54A mutants exhibited reduced half-lives (15 and 18 min, respectively) compared with WT CdnL (32 min) (SI Appendix, Fig. S4A and B and Table S1). These observations are consistent with the CdnL–RNAP interaction contributing to CdnL stability under carbon-starvation conditions. To test the sufficiency of this interaction in determining CdnL stability, we put the V39A and P54A point mutants into a Δ*spoT* background and performed another carbon starvation. If the CdnL–RNAP interaction serves as a barrier to CdnL proteolysis by ClpXP, we expected that these point mutants with a reduced RNAP interaction may still be less stable than WT CdnL in a Δ*spoT* background. However, we found that the point mutants are equally as stable as WT CdnL in the Δ*spoT* background (SI Appendix, Fig. S4C and D). These results indicate that disrupting the RNAP–CdnL interaction is not sufficient to promote CdnL clearance during starvation and reiterates the dependency on SpoT to stimulate CdnL turnover.

### CdnL is cleared during the stationary phase in a SpoT- and ClpXP-independent manner

In addition to starvation, another time in which cells experience nutrient stress is during the stationary phase when resources are depleted as a population saturates. (p)ppGpp levels peak upon entry into the stationary phase, and then dip to stabilize at levels that are higher than basal levels occurring during logarithmic growth (28). Levels of mycobacterial and *Rhodobacter sphaeroides* CdnL homologs, which are called CarD, were shown to decrease during the stationary phase (22, 29). The potential connection between elevated (p)ppGpp levels and clearance of CdnL during the stationary phase has not yet been examined. As we have found that both SpoT and ClpXP play important roles in reducing CdnL levels during starvation when (p)ppGpp levels are high, we were curious to understand how these factors might contribute to regulating CdnL during the stationary phase in *Caulobacter*. To this end, we assessed CdnL protein levels from mid-log to late stationary phase in WT, CdnLDD, and Δ*spoT* strains (SI Appendix, Fig. S5). CdnLDD levels were consistently higher than CdnL levels in both WT and Δ*spoT*, with Δ*spoT* having the lowest CdnL levels throughout. In all strains, CdnL levels started to decline at an OD_600_ of about 1.0. This reduction was stark for both WT and Δ*spoT*, while it was more gradual for CdnLDD. Surprisingly, we found that CdnL was cleared in all strains by an OD_600_ of about 1.6. Because CdnLDD was cleared, this suggests that regulation of CdnL during stationary phase is ClpXP independent, which is different from the regulation of *Mycobacterium smegmatis* CarD (22). Additionally, since SpoT is the sole generator of (p)ppGpp in *Caulobacter*, this demonstrates that regulation of CdnL during stationary phase is (p)ppGpp independent and, therefore, distinct from that of carbon starvation.

### CdnL and CdnLDD exhibit reduced chromosomal binding during starvation

Mechanistically, we have shown that CdnL is cleared posttranscriptionally in a SpoT- and ClpXP-dependent manner, and mutation of the C-terminal degron to CdnLDD stabilizes protein levels during starvation. As CdnL is a transcription factor, we sought to understand whether stabilization of CdnL protein would permit increased CdnL occupancy on the chromosome during starvation, presumably via interaction with RNAP, thus leading to disadvantageous transcriptomic changes during carbon starvation. To begin to understand the consequences of CdnL regulation on transcriptional reprogramming during nutrient limitation, we used chromatin immunoprecipitation sequencing (ChIP-seq) to assess the occupancy of WT CdnL and CdnLDD on the chromosome under both nutrient-replete (M2G) conditions and after 60 min of carbon starvation. We selected this time point as WT CdnL was almost completely cleared (Fig. 1B and C), and broad transcriptional changes are reported at this point (23).

**Fig. 3.**
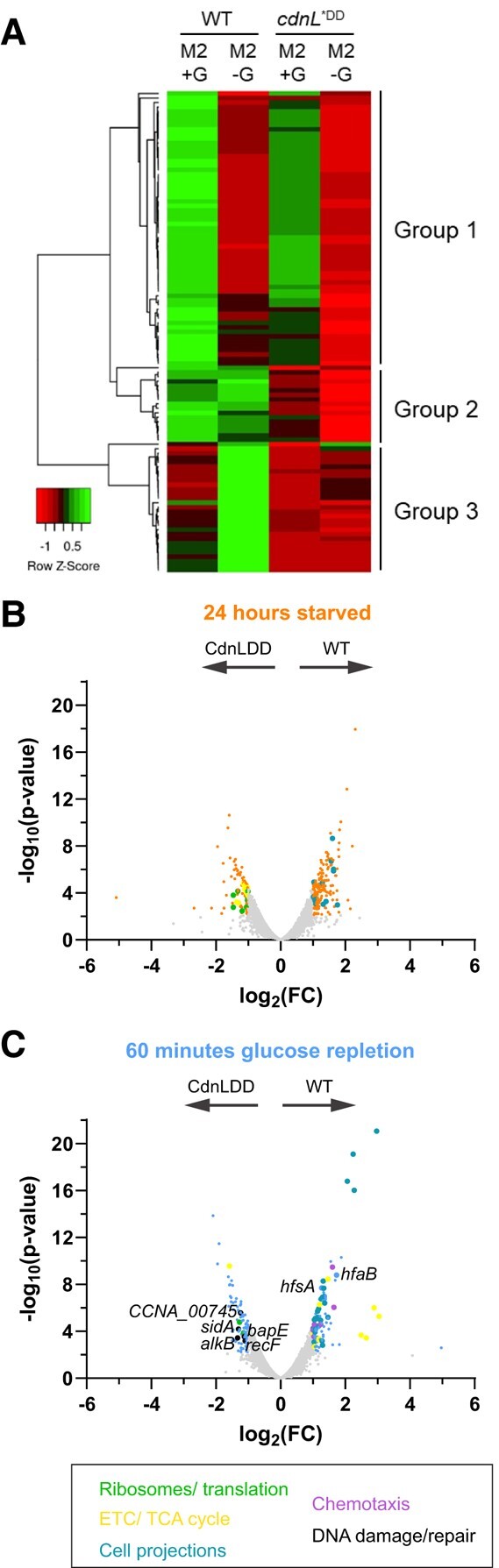
CdnL stabilization impacts transcription of ribosome-related genes. A) Heat map of CdnL/CdnLDD binding across the chromosome in M2G and 60 min of glucose starvation in M2. Green indicates high relative binding, while red indicates low relative binding. Group 1 shows genomic loci with a reduction of WT CdnL binding after starvation in M2. Group 2 shows genomic loci with equal WT CdnL binding after starvation in M2. Group 2 shows genomic loci with an increase in WT CdnL binding after starvation in M2. Row *Z*-score = (CdnL Fold-enrichment value in the sample of interest − mean CdnL Fold enrichment across all samples)/SD. B) Volcano plot comparing differences in gene expression between WT and CdnLDD at 24 h of starvation, as measured by RNA-seq. Negative log_10_ of the *P*-value is plotted against log_2_ of the fold change mRNA counts in WT vs. CdnLDD (FC = WT mRNA counts/CdnLDD mRNA counts). Orange points indicate transcripts with FDR < 0.05 and |log_2_(FC)| > 1, while gray points indicate transcripts that are not significantly different. Teal points indicate transcripts associated with cell projections, such as pili and flagella, while yellow points indicate transcripts associated with the ETC and TCA cycle. Arrows indicate direction of higher expression for respective strains. C) Volcano plot comparing differences in gene expression between WT and CdnLDD at 0 min of starvation, as measured by RNA-seq. Negative log_10_ of the *P*-value is plotted against log_2_ of the fold change mRNA counts in WT vs. CdnLDD (FC = WT mRNA counts/CdnLDD mRNA counts). Light blue points indicate transcripts with FDR < 0.05 and |log_2_(FC)| > 1, while gray points indicate transcripts that are not significantly different. Purple points indicate transcripts associated with chemotaxis, while black points indicate transcripts associated with DNA damage and repair. Arrows indicate direction of higher expression for respective strains.

After identifying 107 peaks with a >2-fold enrichment in the binding profile of WT CdnL in M2G compared with the Δ*cdnL* control, we looked at these peaks in the binding profiles of WT CdnL and CdnLDD in both M2G and M2 (SI Appendix, Fig. S6 and Dataset S1). We observed three clusters of loci with distinct CdnL association profiles. Consistent with our previous transcriptomic analyses, DAVID analysis of locus function revealed a significant enrichment in aminoacyl tRNA biosynthesis and ribosomal genes for all three groups (Dataset S1) (14). In M2G, the binding profiles of WT CdnL and CdnLDD for groups 1 and 3 were well correlated with each other and with our prior ChIP-seq analysis of CdnL association across the genome (Fig. 3A) (14). Group 2 genomic loci showed little to no binding of CdnLDD, while WT CdnL showed high relative binding (Fig. 3A). While this could indicate that mutation to the C-terminus of CdnL impacts DNA binding in nutrient-rich conditions, we observed no growth or morphological defects for CdnLDD, suggesting that these alterations in CdnL binding are not physiologically meaningful when nutrients are available.

During carbon starvation (M2 media), WT CdnL had an overall reduction in binding at group 1 genomic loci, while groups 2 and 3 showed either equal or increased CdnL binding (Fig. 3A). This is surprising given that CdnL protein levels are significantly reduced during starvation (Fig. 1B and C). It is possible that low levels of CdnL protein remain and are important for gene regulation at these loci, as recent studies have suggested that mycobacterial CarD can also function as a repressor and *R. sphaeroides* CarD negatively regulates its own promoter (30–33). Interestingly, CdnLDD showed an overall decrease in binding for all three groups after 60 min of starvation even though CdnLDD remains present during starvation (Fig. 3A). These observations suggest that CdnLDD largely does not associate with the DNA after 60 min of carbon starvation in spite of its stability and imply additional layers of CdnL regulation outside of controlling CdnL protein levels.

### WT and CdnLDD have similar transcriptional profiles after 60 min of starvation

Although CdnLDD does not stably interact with the chromosome after 60 min of carbon starvation, we were interested in understanding whether CdnLDD could still facilitate transcriptional changes. To address this, we used RNA-sequencing (RNA-seq) to assess the transcriptional profiles of WT and CdnLDD at 0 and 60 min of carbon starvation. In support of prior studies, large-scale transcriptional changes were observed for both WT and CdnLDD over 60 min of starvation (Datasets S2 and S3) (23). To gain an understanding of the putative direct targets of CdnL that are impacted by starvation, we compared the promoter regions associated with the 107 peaks identified by ChIP-seq to our RNA-seq analyses for genes differentially regulated in WT or CdnLDD between 0 and 60 min of starvation. WT CdnL immunoprecipitated 52 of the corresponding promoters out of 1,455 differentially expressed genes, while CdnLDD immunoprecipitated 53 of these promoters out of 1,493 differentially expressed genes. Of the 52 putative direct WT CdnL targets that change during starvation, 36 are also direct targets of CdnLDD. These observations suggest that most of the transcriptional changes that occur during carbon starvation are indirectly related to CdnL. This agrees with our previous results, which suggested that the majority of CdnL targets are indirect (14). Similarly, the majority of differentially expressed genes were not associated with *M. smegmatis* CarD binding (32). Nonetheless, here we find that approximately half of *Caulobacter* CdnL's direct targets identified by ChIP-seq changed in abundance during starvation.

In comparing the RNA-seq datasets from CdnLDD to WT at 0 min carbon starvation, we found that 101 genes were at least 2-fold differentially regulated, again indicating that altering the CdnL C-terminus may impact CdnL function (SI Appendix, Fig. S6B and Dataset S4). However, CdnLDD cells do not exhibit an obvious phenotype, further suggesting that any transcriptional changes caused by CdnLDD are not sufficient to impact fitness under nutrient-replete conditions. DAVID functional annotation analyses pointed to an enrichment of transcripts related to the ribosome in CdnLDD, which reinforces our ChIP-seq data and previous transcriptomic analyses implicating CdnL in the regulation of anabolic genes (SI Appendix, Fig. S6B; Datasets S1 and S4) (14). At 60 min of starvation, the number of genes at least 2-fold differentially regulated between WT and CdnLDD was reduced to 71 (SI Appendix, Fig. S6C and Dataset S5). These relatively small differences in the transcriptional profiles between WT and CdnLDD are consistent with their generally similar binding profiles demonstrated by ChIP-seq and further suggest that CdnL stabilization does not cause large-scale changes in transcription after 60 min of starvation.

### CdnL stabilization impacts transcription after 24 h of starvation and upon outgrowth

While CdnL stabilization does not greatly impact the immediate transcriptional response to starvation, *Caulobacter* likely experiences longer periods of nutrient limitation in nature. We, therefore, assessed transcriptional changes in WT and CdnLDD strains after 24 h in the absence of a carbon source. Indeed, we found that the WT transcriptome changes from 60 min to 24 h of starvation, prompting us to investigate potential differences between WT and CdnLDD transcripts at additional time points (Dataset S6).

We decided to assess the WT and CdnLDD transcriptional profiles at 24 h of starvation, a time point in which CdnLDD remains stable (SI Appendix, Fig. S7A). At this time point, we found 279 genes to be 2-fold differentially regulated between WT and CdnLDD, which is ∼2.5- and 4-fold greater than the number of genes differentially regulated at 0 and 60 min of starvation, respectively (Fig. 3B and Dataset S7). For the genes more highly expressed in WT compared with CdnLDD, DAVID functional annotation analyses revealed a relative up-regulation of transcripts associated with cell projections, such as flagella and pili (Fig. 3B and Dataset S7). This suggests that for WT, the cell cycle was retained in the swarmer cell phase, which is consistent with (p)ppGpp acting to slow the swarmer-to-stalked transition (2).

Of the genes up-regulated in CdnLDD compared with WT, many were associated with ribosomes, including translation initiation and elongation factors (Fig. 3B and Dataset S7). While we also saw an up-regulation of ribosome-related transcripts in CdnLDD at 0 min of starvation, higher than normal levels may be particularly problematic when cells are enduring long periods of starvation. Indeed, the production of ribosomes, and protein synthesis in general, is typically reduced under starvation conditions to slow growth and allow resources to be diverted to amino acid biosynthesis (34–36). Additionally, DAVID analyses pointed to an enrichment of transcripts associated with metabolic pathways, such as the electron transport chain (ETC) and tricarboxylic acid (TCA) cycle, in CdnLDD at 24 h of starvation, suggesting increased flux through these pathways (Fig. 3B and Dataset S7).

We also wanted to understand whether CdnL stabilization could affect transcription during the adaptation phase when nutrients are replenished after a long starvation. We, therefore, assessed the transcriptome of WT and CdnLDD 60 min after the addition of glucose, a time point in which WT CdnL has not yet returned to basal levels (SI Appendix, Fig. S7B). After replenishing glucose and allowing the cells to recover for 60 min, 199 genes were found to be differentially regulated, which is almost 2- and 3-fold greater than the number of genes differentially regulated at 0 and 60 min of starvation, respectively (Fig. 3C and Dataset S8). In addition to again finding a relative up-regulation of transcripts associated with cell projections, DAVID analyses also showed an enrichment of transcripts for genes involved in chemotaxis and oxidative phosphorylation in WT (Fig. 3C and Dataset S8). Transcripts associated with holdfast synthesis (*hfsA*) and attachment (*hfaB*) were also up-regulated (Fig. 3C and Dataset S8). Together, these observations suggest that while WT cells were responding to replenished nutrient availability by resuming growth, cell cycle progression, and polar development, CdnLDD cells may be delayed. For CdnLDD, ribosome-related transcripts were still found to be up-regulated (Fig. 3C and Dataset S8). Interestingly, additional transcripts enriched in CdnLDD point to an up-regulation of genes involved in the DNA damage response and repair pathways, including the alkylated DNA repair protein *alkB* (CCNA_00009), the SOS-induced inhibitor of cell division *sidA* (CCNA_02004), an oxidative DNA demethylation family protein (CCNA_00745), the bacterial apoptosis endonuclease *bapE* (CCNA_00663), and the DNA replication and repair protein *recF* (CCNA_00158; Fig. 3C and Dataset S8). Up-regulation of these transcripts could suggest DNA damage in CdnLDD.

These RNA-seq analyses reveal that CdnL stabilization causes increased levels of transcripts associated with ribosomal and protein synthesis genes under both nutrient-rich and carbon-starved conditions, as well as an increase in transcripts associated with the ETC and TCA after 24 h of starvation. During nutrient repletion, CdnL stabilization leads to an up-regulation of transcripts related to DNA damage response and repair proteins, potentially indicating an increase in DNA damage.

### CdnL clearance during starvation is necessary for efficient outgrowth

**Fig. 4.**
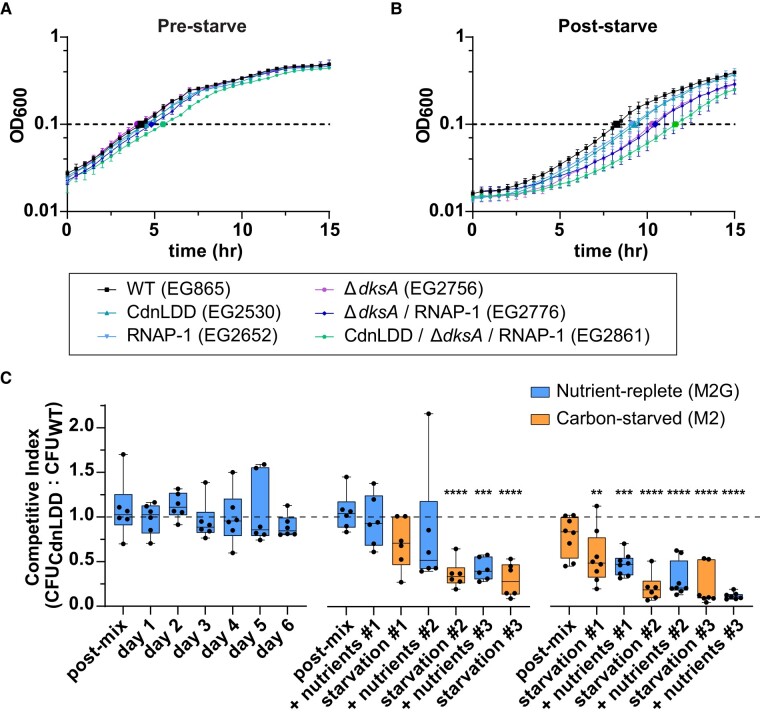
Efficient outgrowth after starvation requires SR-mediated transcriptional reprogramming. A) Growth curve in M2G minimal media prior to 24 h of starvation. Error bars represent ±1 SD of three biological replicates. B) Growth curve in M2G minimal media following 24 h of starvation. Error bars represent ±1 SD of three biological replicates. C) Box-and-whisker plot of the ratio of CdnLDD to WT CFU ratio during competition. Dashed line indicates a ratio of 1, when number of CdnLDD colonies = number of WT colonies. Postmix and prestarve indicate sample taken just after mixing the strains and prior to the initial incubation. Line within each box indicates median of six biological replicates (three mixed cultures in two combinations; for more detail, see Methods). Whiskers show minimum to maximum value. Box encases 25th to 75th percentiles. Statistical analysis uses ANOVA (Dunnett's multiple comparisons test) comparing each mean to the mean of the first postmix sample in the nutrient-replete competition (far left). No asterisk indicates the mean of that sample is not significantly different. **P < 0.005; ***P < 0.0005; ****P < 0.0001.

Since we observed transcriptional changes between WT and CdnLDD during starvation and upon nutrient repletion, we wondered whether CdnL stabilization impacts the ability of cells to adapt to nutrient fluctuations. As increased protein synthesis and metabolic activity during starvation are wasteful, and DNA damage can be deadly, we suspected that CdnLDD cells had physiological defects after longer starvation and when adapting to nutrient repletion. We, therefore, hypothesized that CdnL clearance during starvation facilitates transcriptional changes that enable adaptation by ultimately promoting survival instead of anabolism and proliferation. To this end, we measured the growth of WT and CdnLDD strains both before a 24-h carbon starvation and after the 24-h starvation upon glucose repletion. Before starvation, WT and CdnLDD showed similar growth rates, with doubling times of 2.1 and 2.2 h, respectively (Fig. 4A; SI Appendix, Table S2). During the outgrowth from 24 h of starvation, the strains again had similar growth rates; however, CdnLDD had a longer lag time compared with WT and reached an OD of 0.1 after about 9.3 h as opposed to 8.3 h, respectively (Fig. 4B; SI Appendix, Table S2). This is consistent with our observations from RNA-seq indicating that growth, cell cycle progression, and polar development are delayed for CdnLDD cells in the 60 min following glucose repletion (Fig. 3C and Dataset S8).

Because we observed that cells with an inability to clear CdnL have a defect in outgrowth from carbon starvation, we wondered how this phenotype might relate to the effects of other transcriptional regulators involved in the SR. We repeated these experiments, this time assessing the growth of several mutant strains, including: a strain expressing an RNAP with a (p)ppGpp-binding site 1 mutation (RNAP-1) (11); a strain lacking the transcription factor DksA (Δ*dksA*), which normally would bind to RNAP to form the second (p)ppGpp-binding pocket during starvation (9, 12); a Δ*dksA*/RNAP-1 strain, thus generating an RNAP that is essentially blind to the major effects of (p)ppGpp; and a CdnLDD/Δ*dksA*/RNAP-1 strain. Before starvation, the RNAP-1, Δ*dksA*, and Δ*dksA*/RNAP-1 strains all exhibited growth rates very close to those of WT and CdnLDD, while the CdnLDD/Δ*dksA*/RNAP-1 strain had a slightly longer doubling time and lag time (Fig. 4A; SI Appendix Table S2). After the 24-h starvation, there was a clear striation in outgrowth, with the RNAP-1 strain displaying a similar lag time to CdnLDD, followed by Δ*dksA* and Δ*dksA*/RNAP-1 ∼1 h later. The CdnLDD/Δ*dksA*/RNAP-1 strain showed the greatest lag upon outgrowth, reaching an OD of 0.1 after about 11.6 h in comparison with 8.3 h for WT (Fig. 4B; SI Appendix, Table S2). These data indicate that clearance of CdnL is of similar importance during adaptation as DksA and binding of (p)ppGpp to RNAP site 1. We conclude that the ability of *Caulobacter* to efficiently adapt to nutrient repletion following a period of starvation requires appropriate SR-mediated transcriptional reprogramming, which includes the combined effects of (p)ppGpp binding to RNAP, DksA, and clearance of CdnL.

### CdnL clearance during starvation is important for adaptation to nutrient limitation during competition

On its own, CdnL stabilization caused a 1-h delay in growth during adaptation to changes in nutrient status (Fig. 4B; SI Appendix, Table S2). We also observed transcriptional changes suggesting that CdnL stabilization promotes the transcription of genes that are typically down-regulated upon SR activation and causes cells to accrue DNA damage upon outgrowth (Fig. 3B and C; Datasets S7 and S8). Because of these observations, we wondered whether cells with a stabilized CdnL would be at a disadvantage if they were forced to compete with WT cells while adapting to changes in carbon availability. We tested this by competing antibiotic-marked CdnLDD cells with WT cells containing a different antibiotic marker. This allowed us to ask whether the presence of CdnL is detrimental to fitness during starvation when in competition with cells that can effectively clear CdnL upon activation of the SR. We mixed equivalent OD units of the strains in either M2G for nutrient-replete conditions or M2 for carbon-starved conditions. Immediately after mixing and every 24 h thereafter, we took a sample of the mixed culture and plated it on solid agarcontaining the appropriate antibiotics in order to compare colony forming units (CFUs) between the CdnLDD and WT strains. We then either diluted the remaining culture into M2G to keep the cells in an exponential phase of growth, or into M2 to starve the cells.

We first tested the ability of CdnLDD to compete with WT under nutrient-replete conditions only. Over the course of 6 days, CdnLDD and WT formed colonies in ∼1:1 ratio, suggesting that CdnL stabilization does not impact fitness when there are nutrients readily available (Fig. 4C, left). However, after a single period of carbon starvation, CdnLDD began to show signs of a competitive disadvantage, forming ∼30% fewer colonies than WT (Fig. 4C, middle, starvation #1). After an additional round of carbon starvation and recovery, WT almost entirely outcompeted CdnLDD in the mixed culture. This trend was more rapid when the initial media was M2, as CdnLDD was at a competitive disadvantage immediately after mixing and prior to incubation of the mixed culture, forming about 20% fewer colonies (Fig. 4C, right, postmix). The disadvantage was exacerbated after only 24 h in this carbon-starved media, where CdnLDD formed 45% fewer colonies than WT (Fig. 4C, right, starvation #1). These data suggest that stabilization of CdnL puts cells at an immediate and significant disadvantage when trying to adapt to fluctuating nutrient availability and supports the idea that one way in which *Caulobacter* adapts to nutrient stress is through clearance of this transcriptional regulator.

## Discussion

Adapting to environmental challenges such as nutrient deprivation requires efficient changes in transcription in order to down-regulate biosynthetic genes and up-regulate those that promote survival. While the direct binding of the SR alarmone (p)ppGpp to RNAP is a major way in which cells alter the transcriptome, other factors play key roles in allowing for an effective response to starvation. For instance, binding of the transcription factor DksA to RNAP has been shown to enhance the transcriptional effects of (p)ppGpp on RNAP (12); however, it is not only the presence of factors that promote stress responses that enables adaptation to nutrient stress, but also the absence of anabolic regulators that shifts the transcriptional balance from anabolism to survival. In this study, we show that *Caulobacter* CdnL, a CarD-family transcriptional regulator involved in ribosome biosynthesis and anabolism, is regulated upon SR activation (13–15). We find that transcriptional control at the *cdnL* promoter is not sufficient to control CdnL levels, and regulation of CdnL protein occurs posttranslationally in a manner dependent on SpoT, with (p)ppGpp being sufficient for CdnL clearance.

We also show that during the SR, CdnL is regulated at the protein level by the ClpXP protease and mutation of a degradation signal in the CdnL C-terminus stabilizes CdnL levels. It remains unknown how ClpXP is stimulated to degrade CdnL during starvation. In vitro, ClpXP is not activated directly by (p)ppGpp (SI Appendix, Fig. S3B). Since it has been shown that the specificity of a protease can be affected by changing ATP concentrations, it is possible that changes in ATP levels caused by SR activation stimulate ClpXP to degrade CdnL (37). Indeed, (p)ppGpp has been shown to impact ATP levels in vivo, and *E. coli* (p)ppGpp-deficient cells have perturbed ATP levels during all phases of growth (38). Preventing (p)ppGpp production by inactivating SpoT could impact ATP levels such that conditions are not permissive for CdnL turnover by ClpXP. In support of this idea, we see stabilization of CdnL protein in the absence of SpoT activity even though ClpXP was not manipulated (Fig. 1D and E; SI Appendix, Figs. S1A, B and S2). Alternatively, there may be a (p)ppGpp-sensitive adaptor protein involved in facilitating CdnL proteolysis (39). Further studies are needed to elucidate this connection between SR activation and ClpXP activity.

Surprisingly, while stable, CdnLDD does not appreciably bind the chromosome after 60 min of starvation, nor does it globally alter transcription at this time point. As CdnL mostly associates with promoters occupied by σ^70^, CdnLDD binding may be regulated by the same (unknown) mechanisms that down-regulate transcription of σ^70^-dependent genes during starvation (14). Alternatively, the interaction of CdnLDD with RNAP may be sterically hindered by other factors, or the kinetics of transcription initiation during starvation may not be permissive to CdnL binding to RNAP. Regardless of the mechanism, our finding that CdnLDD does not stably associate with the chromosome during starvation suggests multiple mechanisms work in tandem to down-regulate CdnL-dependent transcription under these conditions.

Transcriptional changes that arise from CdnL stabilization are most obvious after 24 h of starvation, where we find misregulation of ribosomal and metabolic genes. How CdnLDD causes these transcriptional differences without associating stably with the chromosome is unknown. It could be that CdnLDD association with the chromosome is restored during longer starvation periods, which was not assessed by ChIP-seq here, or that these interactions are more transient during starvation and not captured by our approach. Alternatively, CdnL may have some unidentified functions outside of its interaction with DNA and RNAP, which is revealed by stabilizing the protein during starvation (21). Nonetheless, we find that clearance of CdnL is physiologically important, as CdnL stabilization causes clear adaptation defects. We propose that the combined and potentially synergistic actions of SpoT and ClpXP during the SR facilitate rapid clearance of CdnL protein. CdnL clearance allows for adaptation by transcriptionally down-regulating ribosome biosynthesis and flux through metabolic pathways, thereby promoting *Caulobacter* survival when nutrients are lacking. When conditions become favorable again, re-introduction of CdnL enables cells to adapt by reactivating biosynthesis and metabolism, thereby allowing growth to resume.

The connection between the SR and the regulation of CdnL and its homologs in diverse bacteria has been a point of confusion. Indeed, transcription of the mycobacterial CdnL homolog, *carD*, was initially shown to be up-regulated during starvation, and CarD depletion was reported to sensitize cells to stressors such as starvation (20). However, recent studies indicate that while *carD* transcript levels increase during starvation in mycobacteria due to stabilization of the transcript by the antisense RNA AscarD, CarD protein levels ultimately decrease through reduced translation and degradation by the Clp protease. Transcription of *ascarD* is proposed to be under control of the stress sigma factor SigF, thus connecting regulation of CarD to nutrient stress. This regulation of CarD was shown to help mycobacterial cells respond to various stresses; however, dependence on the SR was not assessed (22).

Likewise, we find CdnL regulation to be functionally important for *Caulobacter* cells to adapt to the stress of nutrient fluctuations. Cells producing the stabilized CdnL variant, CdnLDD, have a 1-h delay in outgrowth from carbon starvation (Fig. 4B; SI Appendix Table S2). This delay is similar to that of the RNAP-1 strain, which in *E. coli* causes a drastic growth delay when shifted to nutrient-limiting media, suggesting that CdnL clearance and (p)ppGpp binding to RNAP at this site are of equal importance in *Caulobacter* (11). Additionally, deletion of *dksA*, one of the most renowned (p)ppGpp effectors, causes a lag time only 1 h longer than CdnLDD, further indicating the importance of CdnL clearance during starvation (9, 12, 40–42). The growth defect of CdnLDD is further exacerbated in the absence of DksA and (p)ppGpp binding to RNAP, suggesting an additive relationship between CdnL clearance and binding of DksA and (p)ppGpp to RNAP (Fig. 4B; SI Appendix Table S2). We also find that CdnLDD cells are rapidly outcompeted by WT cells when subjected to periods of carbon starvation and outgrowth, indicating that proper CdnL regulation is absolutely critical for cells to adapt to nutrient stress (Fig. 4C).

These adaptation defects are likely caused by the effects of CdnLDD on transcription (Fig. 3B and C). While we found that after 60 min of starvation, CdnLDD does not associate with the chromosome and causes a relatively limited number of transcriptional changes, the effects of CdnL stabilization on transcription become more apparent after 24 h of starvation and during the adaptation phase when nutrients are replenished. CdnL stabilization increases transcription of genes associated with ribosomes and metabolic pathways during nutrient stress. As ribosome biogenesis and maintenance are costly, it would be disadvantageous to promote these processes during times of limited resources. Likewise, promoting metabolic gene expression can lead to increased flux through these pathways during a time when extra metabolic activity would not be favored. Additionally, the redox reactions of the ETC, if inappropriately managed, can create reactive oxygen species (ROS), which can lead to DNA damage (43, 44). As many DNA damage response and repair proteins are up-regulated in CdnLDD 60 min after the addition of glucose following a 24-h starvation, it is tempting to speculate that increased flux through the ETC is creating ROS and causing DNA damage. The repair of damaged DNA coupled with the costly misregulation of ribosomes can put cells with a stabilized CdnL at a disadvantage when trying to adapt to nutrient fluctuations and reinitiate growth.

Regulation of CdnL during stress appears to be a common theme across diverse bacterial phyla. The *Borrelia burgdorferi* CdnL homolog, called LtpA, was found to be produced at 23°C, a condition that has been widely used to mimic *B. burgdorferi* in unfed ticks, while levels during incubation of cells at 37°C as well as during mammalian infection were significantly reduced (45). Deletion of *ltpA* prevented *B. burgdorferi* from infecting mice via tick infection, suggesting that LtpA could be important for *B. burgdorferi*'s survival within the tick vector and/or transmission to the mammalian host (45, 46). Similarly, depletion of the *M. tuberculosis* homolog prevented cells from replicating and persisting in mice (20). *Bacillus cereus* homologs were found to be up-regulated in response to various stressors and were important in the recovery response to heat shock (47–49). Because CdnL homologs are broadly found and have been implicated in promoting adaptation and survival under different conditions, we believe that regulation of this transcription factor is a conserved mechanism enabling bacteria to adapt to stress.

## Methods

### *Caulobacter crescentus* and *E. coli* growth media and conditions

*Caulobacter crescentus* NA1000 cells were grown at 30°C in peptone–yeast extract (PYE) medium or minimal media described below. *E. coli* NEB Turbo (NEB Catalog #C2986K) and Rosetta(DE3)/pLysS cells were grown at 37 and 30°C, respectively, in Luria–Bertani medium. Antibiotics for *Caulobacter* growth were used in liquid (solid) medium at the following concentrations: gentamycin, 1 (5) µg/mL; kanamycin, 5 (25) µg/mL; oxytetracycline, 1 (2) µg/mL; and spectinomycin, 25 (100) µg/mL. Streptomycin was used at 5 µg/mL in solid medium. *E. coli* antibiotics were used in liquid (solid) medium as follows: ampicillin, 50 (100) µg/mL; gentamicin, 15 (20) µg/mL; kanamycin, 30 (50) µg/mL; oxytetracycline, 12 (12) µg/mL; and spectinomycin, 50 (50) µg/mL. Strains and plasmids used in this study are listed in SI Appendix, Table S3.

### Starvations

Cells were grown overnight in 2–4 mL minimal media with appropriate antibiotics. For both carbon and nitrogen starvation, M2G (M2 with 0.2% glucose w/v) was used (50). For phosphate starvation, Hutner-base imidazole-buffered glucose glutamate medium (HIGG) was used (51). The next day, mid-log-phase cells were harvested by centrifugation and washed thrice with nutrient-poor media. For carbon starvation, M2 media (without glucose) was used; for nitrogen starvation, M2G-lacking NH_4_Cl was used; and for phosphate starvation, HIGG-lacking Na_2_HPO_4_–KH_2_PO_4_ was used. After the final wash, cells were resuspended in 5 mL nutrient-limited media, divided into four 1.2 mL cultures, and incubated at 30°C shaking at 225 rpm. OD_600_ was taken every 30 min from 0 to 90 min to take samples for immunoblotting. Three biological replicates were obtained for each strain in each condition.

### RelA′ induction

EG1799 and EG1800 were grown overnight in 5 mL M2G with appropriate antibiotics. The next day, mid-log-phase cells were diluted to OD_600_ = 0.2 in 10 mL M2G with 0.3% xylose (w/v). Cultures were distributed into seven 1.4 mL aliquots and incubated as described above. OD_600_ was taken every 30 min from 0 to 120 min. Three biological replicates were obtained for each strain.

### Transcriptional shut-off

EG3190, EG3193, and EG3194 were grown overnight in 2–4 mL M2 with 0.3% xylose and appropriate antibiotics. The next day, mid-log-phase cells were harvested by centrifugation and washed thrice with M2G (to shut off transcription) or M2 (to shut off transcription and starve cells). After the final wash, cells were resuspended in 5 mL M2G or M2 and incubated as described above. A total of three biological replicates were obtained for each strain.

### ClpX* induction

EG802 and EG865 were grown overnight in 2–4 mL M2G with appropriate antibiotics. The next day, mid-log-phase cultures were diluted to OD_600_ = 0.1–0.25 in 5 mL M2G supplemented with 0.3% xylose (w/v) and grown for 2 h. The starvation was performed as described above. Three biological replicates were obtained for each strain.

### Immunoblotting

Immunoblotting samples were prepared by harvesting 0.5 or 1 mL cells by centrifugation and resuspending pellets in OD_600_/0.003 or OD_600_/0.003 μL 1× sodium dodecyl sulfate (SDS) loading dye, respectively. Equivalent OD units of cell lysate were loaded on an SDS–-polyacrylamide gel electrophoresis (PAGE) gel following cell harvest by centrifugation, lysing 1× SDS loading dye, and boiling for 5–10 min. SDS–PAGE and protein transfer to nitrocellulose membranes were followed using standard procedures. Antibodies were used at the following dilutions: CdnL 1:10,000 (14); MreB 1:10,000 (Régis Hallez, University of Namur); SpmX 1:20,000 (Patrick Viollier, University of Geneva); GFP 1:2,000 (Clonetech Labs, catalog #NC9777966); HRP-labeled α-rabbit secondary 1:10,000 (BioRAD, catalog #170-6515); and/or HRP-labeled α-mouse secondary (Cell Signaling Technology, catalog #7076S). Clarity western electrochemiluminescent substrate (BioRAD, catalog #170-5060) was used to visualize proteins on an Amersham Imager 600 RGB gel and membrane imager (GE).

### Protein purification

CdnL and CdnLDD were overproduced in Rosetta (DE3) pLysS *E. coli* from pEG1129 (His-SUMO-CdnL) and pEG1634 (His-SUMO-CdnLDD), respectively. Cells were induced with 1 mM IPTG for 3 h at 30°C. Cell pellets were resuspended in Column Buffer A (50 mM Tris-HCl pH 8.0, 300 mM NaCl, 10% glycerol, 20 mM imidazole, 1 mM β-mercaptoethanol), flash frozen in liquid nitrogen, and stored at −80°C. To purify the His-SUMO-tagged proteins, pellets were thawed at 37°C, and 10 U/mL DNase I, 1 mg/mL lysozyme, and 2.5 mM MgCl_2_ were added. Cell slurries were rotated at room temperature for 30 min, then sonicated and centrifuged for 30 min at 15,000*×g* at 4°C. Protein supernatants were then filtered and loaded onto a pre-equilibrated HisTrap FF 1 mL column (Cytiva, Marlborough, MA, USA). His-SUMO-CdnL and His-SUMO-CdnLDD were eluted in 30% column buffer B (same as column buffer A but with 1 M imidazole), and peak fractions were concentrated. The His-Ulp1 SUMO protease was added to a molar ratio (protease:protein) of 1:290 and 1:50 for His-SUMO-CdnL and His-Sumo-CdnLDD, respectively, and dialyzed into 1 L column buffer A overnight at 4°C. The cleaved protein solutions were again loaded onto a HisTrap FF 1 mL column. Peak flow-through fractions were combined, concentrated, and applied to a Superdex 200 10/300 GL (Cytiva) column equilibrated with storage buffer [50 mM 4-(2-hydroxyethyl)piperazine-1-ethanesulfonic acid (HEPES)–NaOH pH 7.2, 100 mM NaCl, 10% glycerol]. Peak fractions were combined, concentrated, snap-frozen in liquid nitrogen, and stored at −80°C. ClpX was purified using a similar protocol as described for *E. coli* ClpX (24). Recombinant his-tagged ClpP was purified as described (52).

### In vitro proteolysis

In vitro proteolysis reactions were performed with purified ClpX, ClpP, and CdnL or CdnLDD, as previously described (25).

### Monitoring CdnL protein levels

EG865, EG1139, and EG2530 were inoculated in 2 mL M2G. The next day, the cultures were diluted to an OD_600_ of ∼0.001 and grown for ∼18 h or until the cultures reached an OD_600_ of ∼0.4–0.5. At OD_600_ of ∼0.4–0.5, 500 μL of culture sample was taken to make protein samples for immunoblotting, as described above. Samples were then taken in the following 1, 2, 4, 6, 7, 8, and 25 h. Immunoblotting was performed as described above, except following the transfer, membranes were stained with Ponceau stain for total protein normalization.

### Chromatin immunoprecipitation coupled to deep sequencing

EG865, EG1898, and EG2530 were grown overnight in 6 mL M2G (M2 with 0.2% glucose w/v) at 30°C (shaking at 200 rpm), respectively. The next day, cultures were diluted into 100 mL M2G and grown to an OD_660_ of 0.5. Cells were harvested by centrifugation at 8,000 rpm for 10 min at 25°C, and cell pellets were washed three times with 50 mL of M2 media (without glucose) at 25°C. Washed cell pellets were split into two and used to inoculate 50 mL of M2 (carbon starvation) and 50 mL of M2G (preheated culture medium at 30°C). Cultures were incubated at 30°C (shaking at 200 rpm) for additional 60 min and then, supplemented with 10 μM sodium phosphate buffer (pH 7.6) and treated with formaldehyde (1% final concentration) at room temperature for 10 min to achieve crosslinking. Subsequently, the cultures were incubated for an additional 30 min on ice and washed three times in phosphate-buffered saline (pH 7.4). The resulting cell pellets were stored at −80°C. Chromatin immunoprecipitation for CdnL was performed, as previously described (14).

Immunoprecipitated chromatins were used to prepare sample libraries used for deep sequencing at Fasteris SA (Geneva, Switzerland). ChIP-seq libraries were prepared using the DNA Sample Prep Kit (Illumina) following manufacturer instructions. Single-end run was performed on an Illumina Next-Generation DNA sequencing instruments (NextSeq High), 50 cycles were performed and yielded several million reads per sequenced samples. The single-end sequence reads stored in FastQ files were mapped against the genome of *C. crescentus* NA1000 (NC_011916.1) using Bowtie2 Version 2.4.5 + galaxy1 available on the web-based analysis platform Galaxy (https://usegalaxy.org) to generate the standard genomic position format files (BAM). ChIP-seq reads sequencing and alignment statistics are summarized in Dataset S1. Then, BAM files were imported into SeqMonk version 1.47.2 (http://www.bioinformatics.babraham.ac.uk/projects/seqmonk/) to build ChIP-seq normalized sequence read profiles. Briefly, the genome was subdivided into 50 bp, and for every probe, we calculated the number of reads per probe as a function of the total number of reads (per million, using the Read Count Quantitation option; Dataset S1). Using the web-based analysis platform Galaxy (https://usegalaxy.org), CdnL(M2 + G) ChIP-seq peaks were called using MACS2 Version 2.2.7.1 + galaxy0 (no broad regions option) relative to the *ΔcdnL*(M2 + G) negative ChIP-seq control. The *q*-value (false discovery rate, FDR) cutoff for called peaks was 0.05. Peaks were rank ordered according to their fold-enrichment values (Dataset S1, the 107 ChIP-seq CdnL(M2 + G) statistical peaks with a fold enrichment >2 were retained for further analysis). MACS2 analyzed data illustrated in Fig. S6A are provided in Dataset S1. Then, CdnL(M2 + G) ChIP-seq equivalent peaks (overlapping peak “start” and “end” MACS2 coordinates) were searched in the MACS2 statistical peak analyses of ChIP-seq CdnL(M2-G), ChIP-seq CdnLDD(M2 + G), and ChIP-seq CdnLDD(M2-G) datasets (relative to the *ΔcdnL*(M2 + G) or (M2-G) negatives ChIP-seq controls, accordingly). In cases where no overlapping peaks were identified, a default fold-enrichment value of 1 was assigned. These data were submitted to the http://www.heatmapper.ca/expression/ website to generate a heat map (Parameters: Clustering method “average linkage”; Distance measurement method “Pearson”; Scale “Row *Z*-score”). Row *Z*-score = (CdnL Fold-enrichment value in the sample of interest − Mean CdnL Fold enrichment across all samples)/SD. The analyzed data illustrated in Fig. 3A as well as the list of genes composing each group are provided in Dataset S1. For each group defined during the heat map analysis, the list of genes potentially regulated by CdnL (presence of a CdnL statistically significant peak detected on the gene's promoter region) was submitted to the DAVID website (The Database for Annotation, Visualization and Integrated Discovery; https://david.ncifcrf.gov/home.jsp) to identify enriched biological themes, particularly gene ontology terms (Dataset S1).

### RNA sequencing

For 0- and 60-min starvation samples, cultures of three independent colonies of EG865 and EG2530 were inoculated into 2 mL M2G overnight. The next day, cultures were diluted into 4 mL M2G and grown to an OD_600_ of 0.4–0.6. Cells were prepared for carbon starvation as described above, except after the final wash, cells were resuspended in 4 mL M2 and were split into two 2 mL samples each. One sample for each replicate was incubated at 30°C shaking at 225 rpm for 60 min, while the other was stabilized using RNAprotect Bacteria Reagent (Qiagen, catalog #76506) following manufacturer's instructions. Briefly, 4 mL RNAprotect was added to 15 mL conical tubes, to which the 2 mL culture samples were added. The conical tubes were vortexed for 5 s, incubated at room temperature for 5 min, and then centrifuged for 10 min at 5,000*×g*. The pellets were flash frozen in liquid nitrogen and stored at −80°C. The 60-min starved samples were harvested in the same way.

For 24 h starved and 60 min recovery samples, cultures of three independent colonies were inoculated into 6 mL M2G and grown overnight. Once cultures were at an OD_600_ of 0.4–0.6, cultures were prepared for carbon starvation as described above except after final wash, cells were resuspended in 6 mL M2 and incubated at 30°C shaking at 225 rpm for 24 h. After 24 h, 2 mL of each culture was removed and stabilized using RNAprotect as described above and flash frozen in liquid nitrogen. To the remaining 4 mL of culture, 40 μL of 20% glucose solution was added, and cultures were incubated for 60 min. Recovery samples were stabilized with RNAprotect and flash frozen in liquid nitrogen.

All samples were processed at SeqCenter (Pittsburg, PA, USA). There, samples were DNAse treated with Invitrogen DNAse (RNAse free). Library preparation was performed using Illumina's Stranded Total RNA Prep Ligation with Ribo-Zero Plus kit and 10 bp IDT for Illumina indices and *Caulobacter*-specific rRNA depletion probes. Sequencing was done on a NextSeq2000 giving 2 × 51 bp reads. Quality control and adapter trimming was performed with bcl2fastq (version 2.20.0.445; default parameters). Read mapping was performed with HISAT2 (version 2.2.0; default parameters + “--very-sensitive”) (53). Read quantification was performed using Subread's featureCounts functionality (version 2.0.1; default parameters + ”-Q 20”) (54). Read counts were loaded into R (version 4.0.2; default parameters) and were normalized using edgeR's (version 1.14.5; default parameters) Trimmed Mean of *M*-values algorithm (55). Subsequent values were then converted to counts per million. Differential expression analysis was performed using edgeR's exact test for differences between two groups of negative-binomial counts.

### Growth curves

EG865, EG2530, EG2652, EG2756, EG2776, and EG2861 were grown overnight in 2 mL M2G. The next day, biological triplicates were diluted to OD_600_ = 0.05 in 100 µL M2G in a 96-well plate. A Cytation1 imaging reader (Agilent, Biotek) measured absorbance every 30 min for 36 h with intermittent shaking. The remainder of each 2 mL culture was diluted into 5 mL M2G and incubated overnight. Log-phase cells were harvested by centrifugation at 8,250*×g* for 5 min, washed thrice with M2, resuspended in a total volume of 4.5 mL M2, and incubated for 24 h. Following the starvation, another growth curve in M2G was run as described above. Approximate time to OD = 0.1 was calculated by solving a trendline using averaged triplicates just prior to OD = 0.1 and just after OD = 0.1. Doubling times were calculated using GraphPad Prism software.

### Competitions

EG3402, EG3404, EG3406, and EG3408 were grown overnight in 2 mL PYE with appropriate antibiotics. The next day, overnight cultures were pelleted at 16,300*×g* for 1 min and washed twice with M2G. Following the final wash, cells were resuspended in 10 mL M2G with appropriate antibiotics and grown overnight. The next day, stationary-phase cells were diluted into 50 mL M2G with appropriate antibiotics and allowed to grow a minimum of 6 h (∼2 doublings in M2G) to an OD_600_ of 0.3–0.6. Cells were then synchronized with modifications to the original protocol (56). Briefly, cells were pelleted at 6,000*×g* for 10 min and then resuspended in 1.5 mL cold 1× M2 salts. The resuspension was transferred to 15 mL Corex tubes. A 1.5-mL cold Percoll was added and cells were centrifuged at 15,000*×g* for 20 min at 4°C. Swarmer cells were transferred to 15 mL conicals, 1× M2 salts were added to fill the tube, and cells were pelleted at 10,000*×g* for 5 min. Cell pellets were resuspended in 1 mL 1× M2 salts and centrifuged at 16,300*×g* for 1 min. The resulting pellet was resuspended in M2 for nutrient-poor conditions or M2G for nutrient-rich conditions, and OD_600_ was recorded.

When starting in nutrient-poor (M2) conditions, cultures were equally diluted to OD_600_ = 0.25–0.4 and mixed 1:1 (EG3402:EG3408, EG3404:EG3406) in 5 mL M2 and incubated for 24 h. Prior to incubation, 50 μL of the mixed cultures were serially diluted, and 100 μL of 10^−4^–10^−6^ dilutions were plated on both PYE–spectinomycin and PYE–kanamycin plates. When starting in nutrient-rich conditions, the same procedure was followed except after plating, the mixed cultures were diluted to OD ∼0.0001 and 0.00005 in 5 mL M2G and allowed to grow for 24 h.

Every 24 h, a culture sample was taken for plating as described above. CFUs were counted on plates following a 2-day incubation at 30°C, and a ratio called the competitive index was calculated from the CFUs formed by the CdnLDD strains compared with the WT strains (EG3408/EG3402; EG3406/EG3404) on the plates that yielded the maximum number of countable colonies.

If in nutrient-rich conditions, a volume of cells approximately equal to OD_600_ = 0.25 were pelleted, washed twice with M2, resuspended in 5 mL M2, and incubated for a 24-h starvation. If in nutrient-poor conditions, cells were diluted to an OD ∼0.0003 in 5 mL M2G and incubated for a 24-h recovery. This was repeated for a total of three rounds. The entire process was repeated for a total of four biological replicates when starting in nutrient-poor conditions and three biological replicates when starting in nutrient-rich conditions. For the competition in only nutrient-rich conditions, a similar procedure was followed, but every 24 h cells were diluted to an OD ∼0.00005 in 5 mL M2G for a total of six rounds. The entire process was repeated to obtain three biological replicates.

## Supplementary Material

pgae154_Supplementary_Data

## Data Availability

All data are included in the manuscript and supplementary material. Raw ChIP-seq data are in the Gene Expression Omnibus (GEO) database (GSE249185 series, accession numbers GSM7927858–GSM7927866). Raw RNA-seq data are in the Sequence Read Archive under accession numbers SRR27130076–SRR27130078, SRR27130555–SRR27130557, and SRR27146334–SRR27146351, and are associated with BioProject PRJNA1049818.

## Preprints

This manuscript was posted as a preprint: https://doi.org/10.1101/2023.12.20.572625.
