# Supplementary material for: Regulation of the transcription factor CdnL promotes adaptation to nutrient stress in Caulobacter
Source: PNAS Nexus. 2024 Apr 10;3(4):pgae154. doi: 10.1093/pnasnexus/pgae154 (PMC11034885; doi:10.1093/pnasnexus/pgae154)
Supplement: pgae154_Supplementary_Data [file pgae154_supplementary_data.zip › PNASNEXUS-PNASNEXUS-2024-00183R-s01.docx]

**
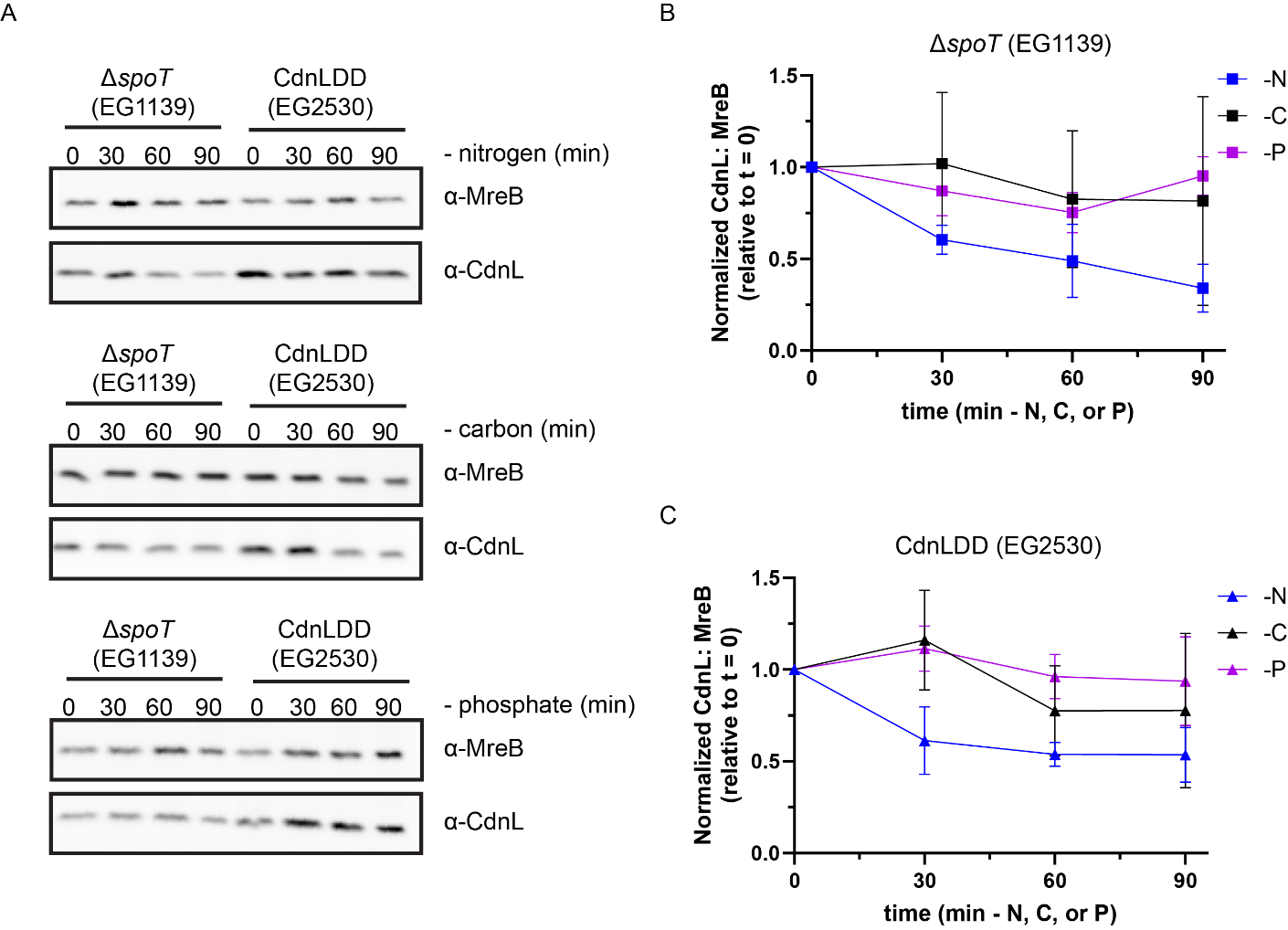
Supplemental Figure S1: CdnL levels are stabilized in Δ*spoT* and CdnLDD**

1. Representative western blot of CdnL during 90 minutes of nitrogen, carbon, and phosphate starvation in Δ*spoT* (EG1139) and CdnLDD (EG2530). Protein samples were taken every 30 minutes. MreB was used as a loading control.
2. Densitometry of CdnL levels (normalized to MreB) relative to t = 0 during nitrogen (-N), carbon (-C), and phosphate (-P) starvations in Δ*spoT* (EG1139) from western blots as performed in A. Error bars represent +/- 1 SD of 3 biological replicates.
3. Densitometry of CdnL levels (normalized to MreB) relative to t = 0 during nitrogen (-N), carbon (-C), and phosphate (-P) starvations in CdnLDD (EG2530) from western blots as performed in A. Error bars represent +/- 1 SD of 3 biological replicates.

**
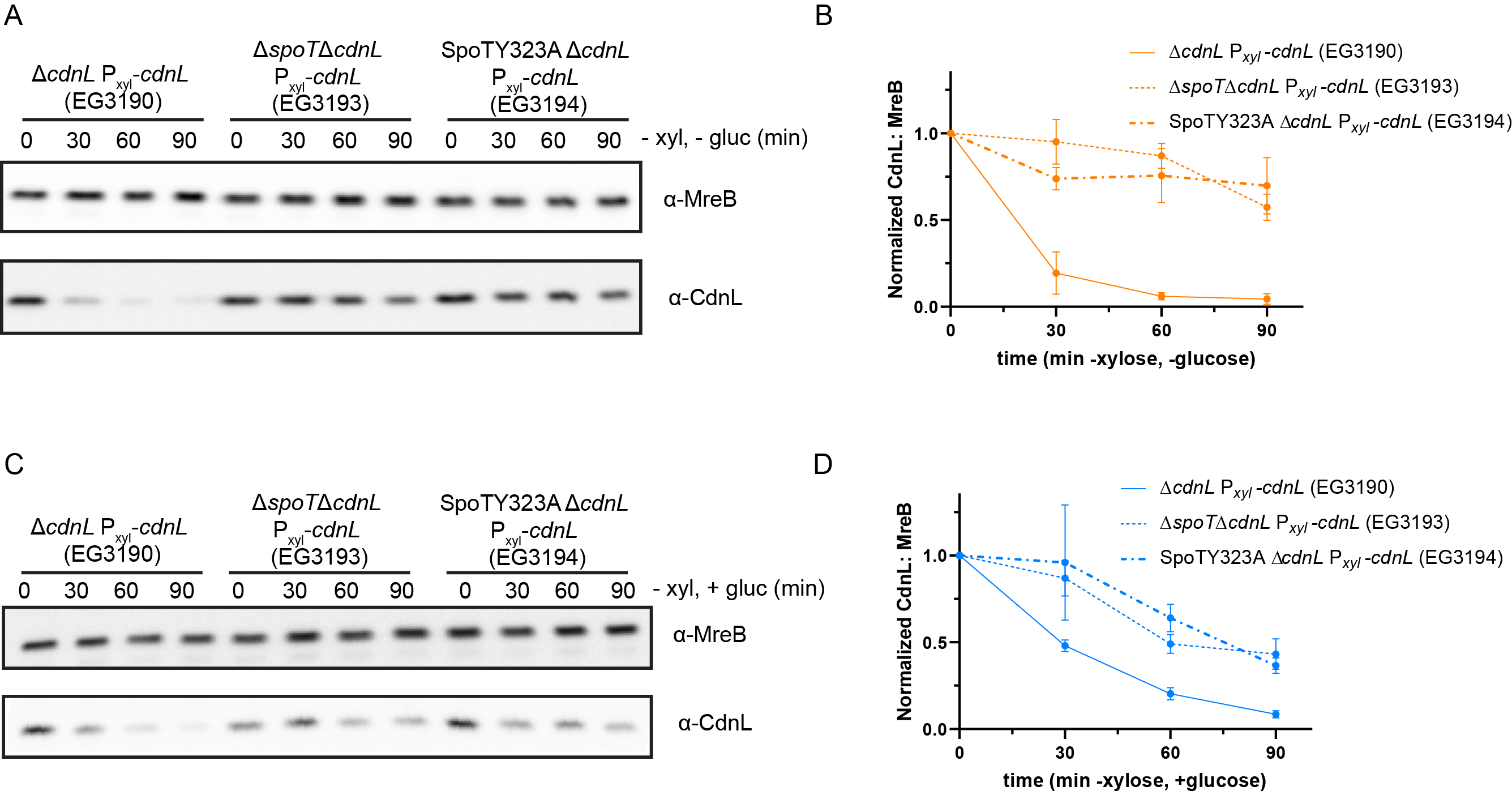
Supplemental Figure S2: Transcriptional regulation of *cdnL* is not sufficient to control CdnL levels**

1. Representative western blot of CdnL during 90 minutes of glucose (gluc) starvation with xylose (xyl) depletion of *cdnL* in a Δ*cdnL,* Δ*spoT*Δ*cdnL*, or SpoTY323AΔ*cdnL* background. Protein samples were taken every 30 minutes. MreB was used as a loading control.
2. Densitometry of CdnL levels (normalized to MreB) relative to t = 0 from western blots as performed in A. Error bars represent +/- 1 SD of 3 biological replicates.
3. Representative western blot of CdnL during xylose (xyl) depletion of *cdnL* in a Δ*cdnL,* Δ*spoT*Δ*cdnL*, or SpoTY323AΔ*cdnL* background. Protein samples were taken every 30 minutes. MreB was used as a loading control.
4. Densitometry of CdnL levels (normalized to MreB) relative to t = 0 from western blots as performed in C. Error bars represent +/- 1 SD of 3 biological replicates.

**
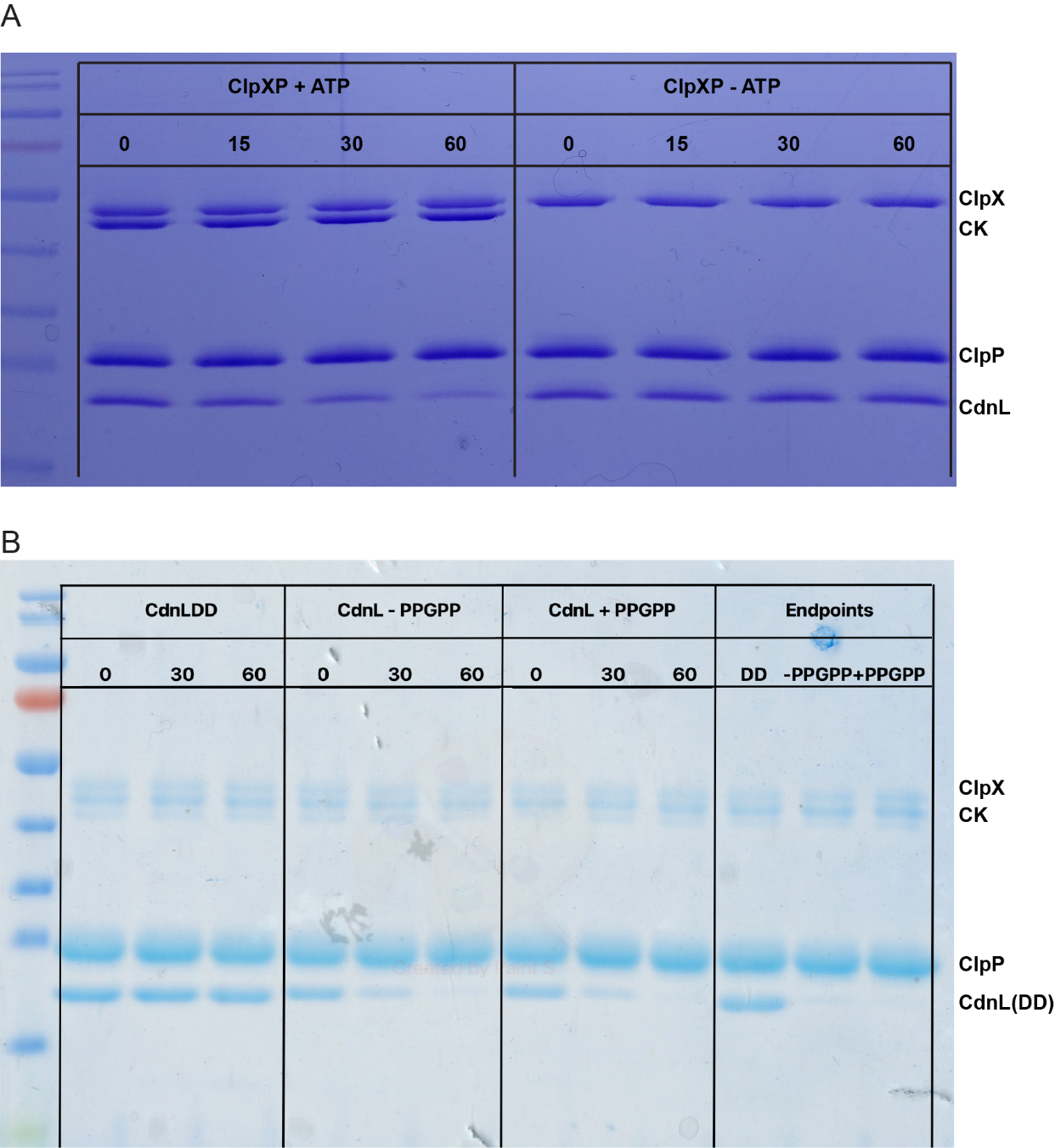
**

**Supplemental Figure S3: CdnL degradation *in vitro* is ATP-dependent and (p)ppGpp does not stimulate ClpXP degradation of CdnL**

1. Representative gel for *in vitro* degradation of CdnL. CK indicates creatine kinase, which is used for ATP regeneration in the reaction. Experiments were performed twice each. Half-live values are 29 min + ATP and > 500 min – ATP.
2. Representative gel for *in vitro* degradation of CdnL or CdnLDD. Protein samples were taken at indicated time points. CK indicates creatine kinase, which is used for ATP regeneration in the reaction. Experiments were performed twice each. Half-life values are 29 min – ppGpp and 28 min + ppGpp.

**
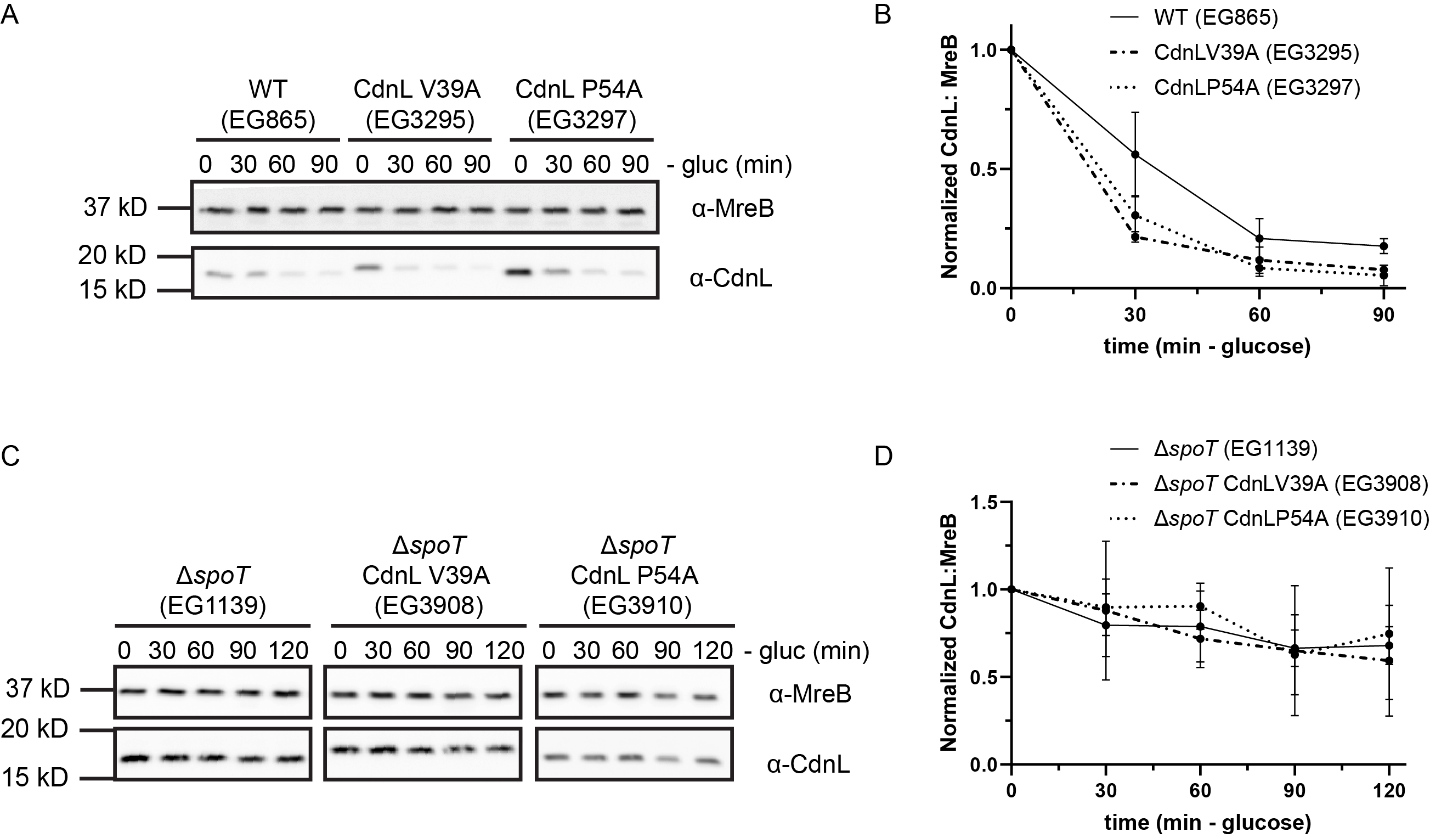
Supplemental Figure S4: CdnL mutants with a reduced interaction with RNAP are cleared more rapidly than WT CdnL**

1. Representative western blot of WT CdnL, CdnL V39A, or CdnL P54A during 90 minutes of glucose (gluc) starvation. Protein samples were taken every 30 minutes. MreB was used as a loading control.
2. Densitometry of WT CdnL, CdnL V39A, or CdnL P54A levels (normalized to MreB) relative to t = 0 from western blots as performed in A. Error bars represent +/- 1 SD of 3 biological replicates.
3. Representative western blot of WT CdnL, CdnL V39A, or CdnL P54A in a Δ*spoT* background during 90 minutes of glucose (gluc) starvation. Protein samples were taken every 30 minutes. MreB was used as a loading control.
4. Densitometry of GFP-AA levels (normalized to SpmX) relative to t = 0 from western blots as performed in A. Error bars represent +/- 1 SD of 3 biological replicates.

**
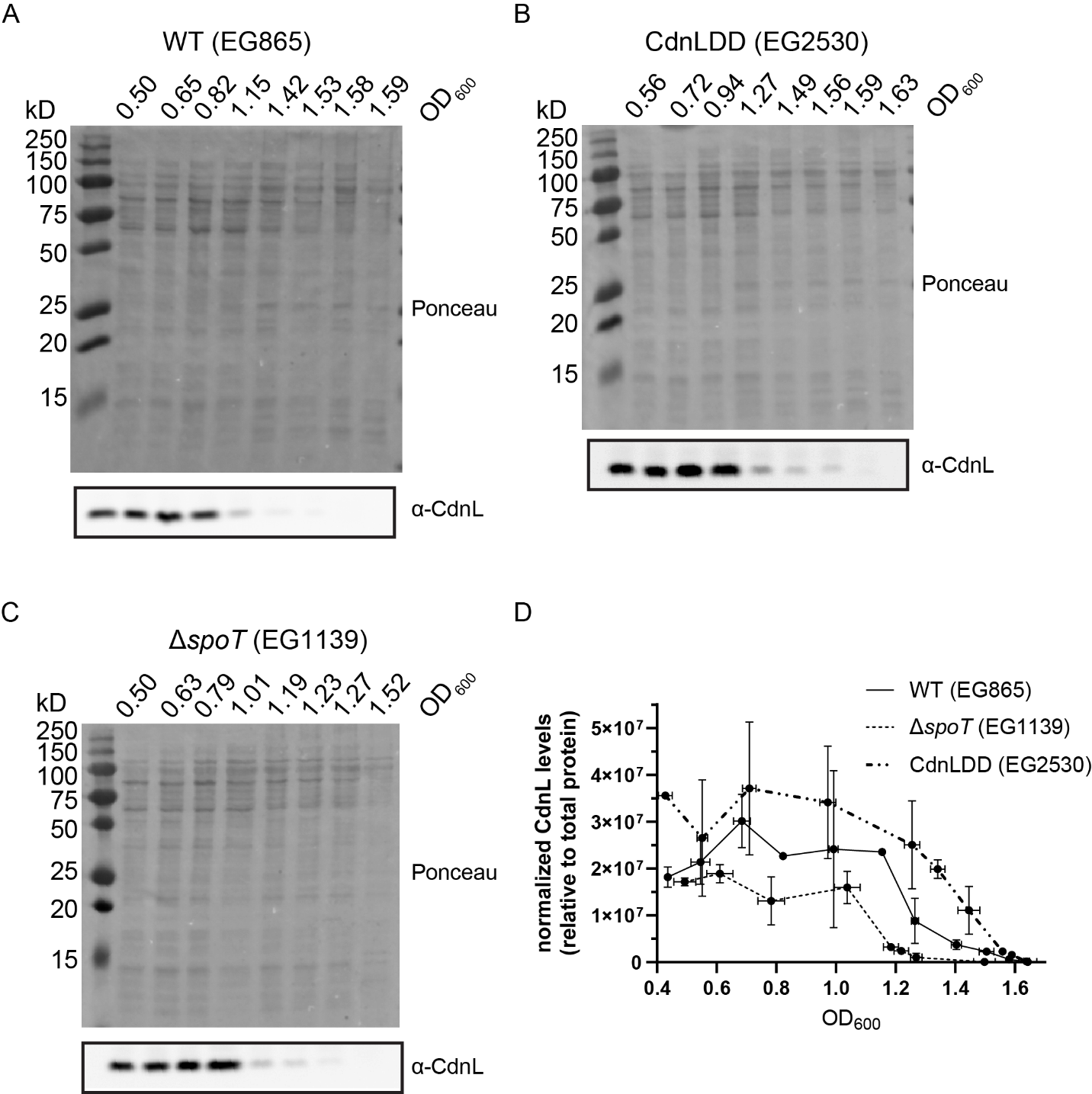
**

**Supplemental Figure S5: CdnL clearance during stationary phase is SpoT- and ClpXP-independent**

1. Representative Ponceau stain and western blot for CdnL in WT (EG865). Samples were taken once OD_600_ = 0.4 – 0.6 for t = 0, and then at t = 1, 2, 4, 6, 7, 8, and 25 hours later. OD_600_ was recorded at each timepoint.
2. Representative Ponceau stain and western blot for CdnL in Δ*spoT* (EG1139). Samples were taken once OD_600_ = 0.4 – 0.6 for t = 0, and then at t = 1, 2, 4, 6, 7, 8, and 25 hours later. OD_600_ was recorded at each timepoint.
3. Representative Ponceau stain and western blot for CdnL in CdnLDD (EG2530). Samples were taken once OD_600_ = 0.4 – 0.6 for t = 0, and then at t = 1, 2, 4, 6, 7, 8, and 25 hours later. OD_600_ was recorded at each timepoint.
4. Densitometry analysis of CdnL/CdnLDD (normalized to total protein) for WT, Δ*spoT,* or CdnLDD strains from OD_600_ ~ 0.4 – 1.6 using Ponceau-stained membranes and western blots as performed in A - C. CdnL levels for OD_600_ values within 0.1 units were averaged. X and Y error bars represent +/- 1 SD of 1 – 3 biological replicates per point.

**
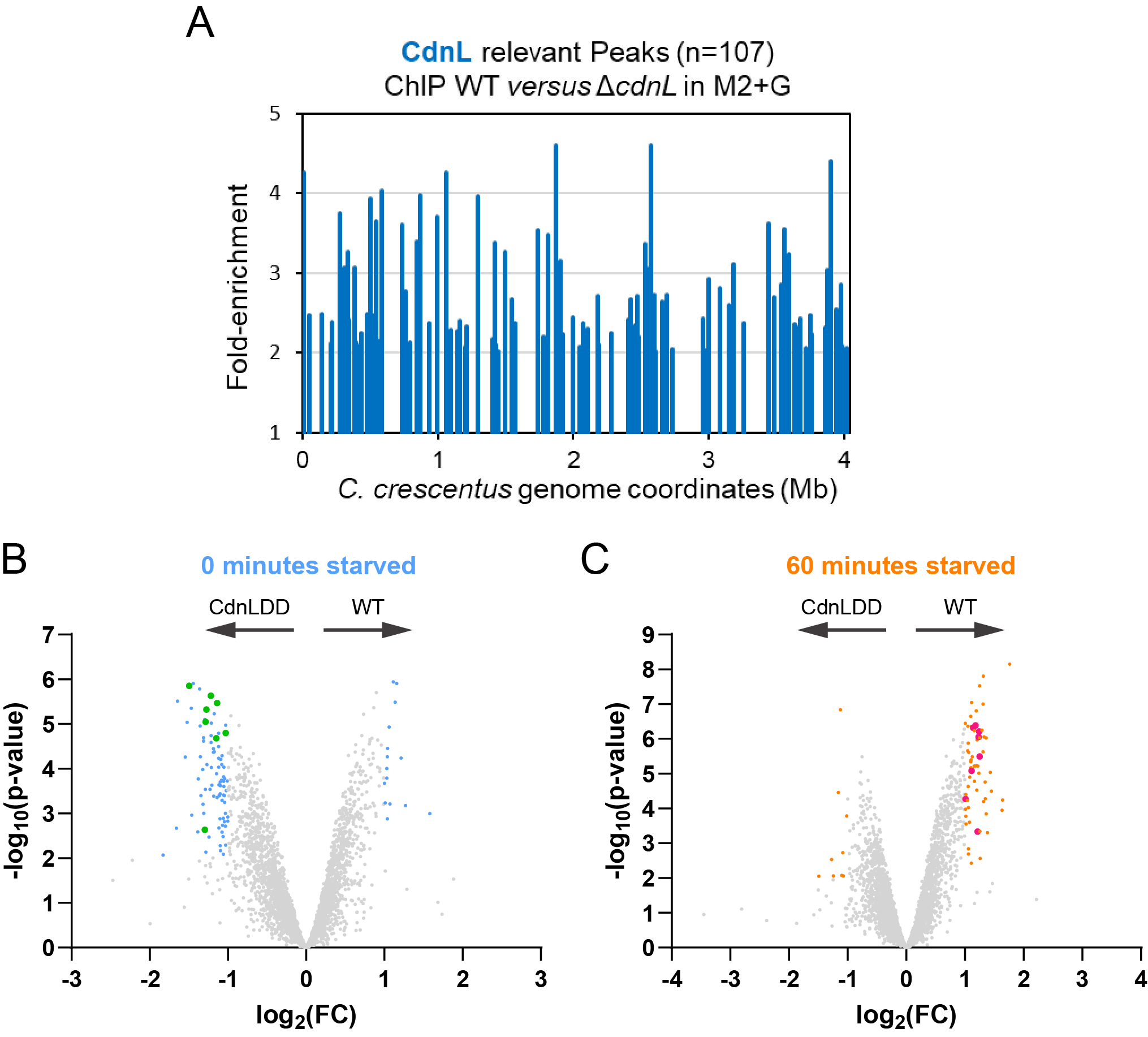
**

**Supplemental Figure S6: CdnL relevant peaks identified by ChIP-seq and transcriptional changes over 60 minutes of starvation**

1. 107 peaks identified by ChIP-seq as having a > 2-fold enrichment compared to the ∆*cdnL* (EG1898) control in M2G. These peaks were selected for further analyses in comparing WT (EG865) to CdnLDD (EG2530) in M2G and M2.
2. Volcano plot comparing differences in gene expression between WT and CdnLDD at 0 minutes of starvation, as measured by RNAseq. Negative log_10_ of the p-value is plotted against log_2_ of the fold change mRNA counts in WT vs CdnLDD (FC = WT mRNA counts/ CdnLDD mRNA counts). Light blue points indicate transcripts with FDR < 0.05 and |log_2_(FC)| > 1, while gray points indicate transcripts that are not significantly different. Green points indicate transcripts associated with ribosomes and translation. Arrows indicate direction of higher expression for respective strains.
3. Volcano plot comparing differences in gene expression between WT and CdnLDD at 60 minutes of starvation, as measured by RNAseq. Negative log_10_ of the p-value is plotted against log_2_ of the fold change mRNA counts in WT vs CdnLDD (FC = WT mRNA counts/ CdnLDD mRNA counts). Orange points indicate transcripts with FDR < 0.05 and |log_2_(FC)| > 1, while gray points indicate transcripts that are not significantly different. Pink points indicate transcripts associated with toxin-antitoxin (TA) systems and stress responses. Arrows indicate direction of higher expression for respective strains.

**
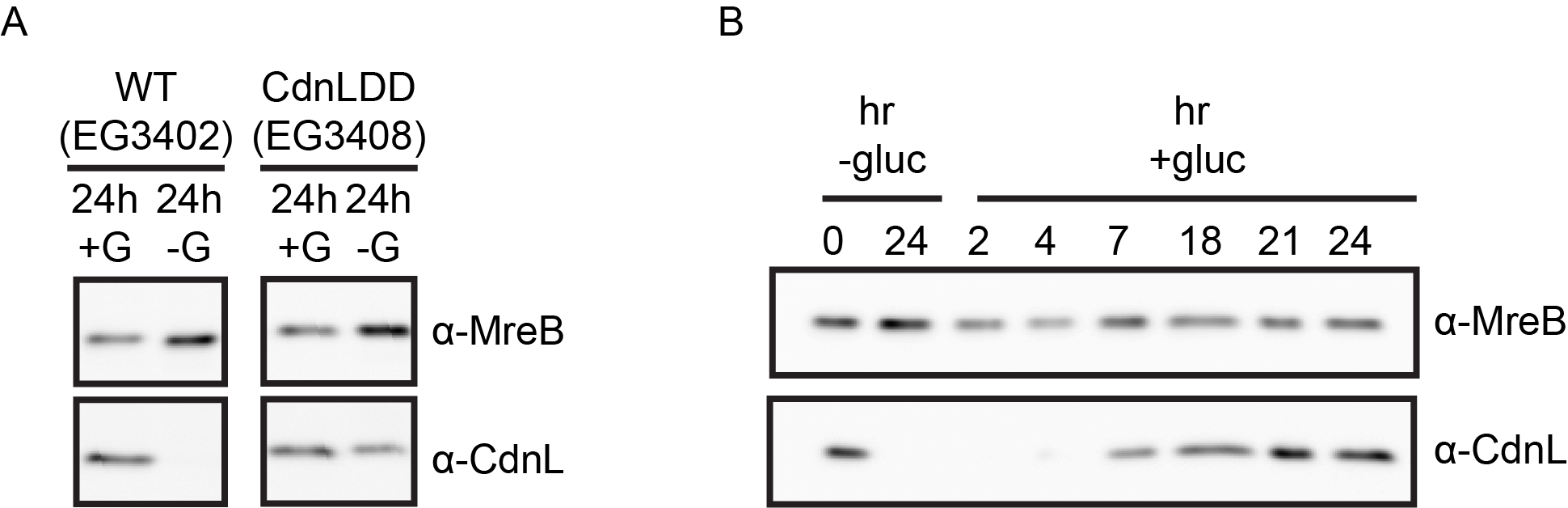
**

**Supplemental Figure S7: CdnLDD is stable after 24 hours of starvation**

1. Representative western blot of WT (EG3402) and CdnLDD (EG3408) before (24h +G) and after (24h -G) 24 hours of starvation. MreB was used as a loading control.
2. Representative western blot of CdnL levels in WT (EG3402) after 24 hours of starvation and at indicated timepoints following the addition of glucose. MreB was used as a loading control.


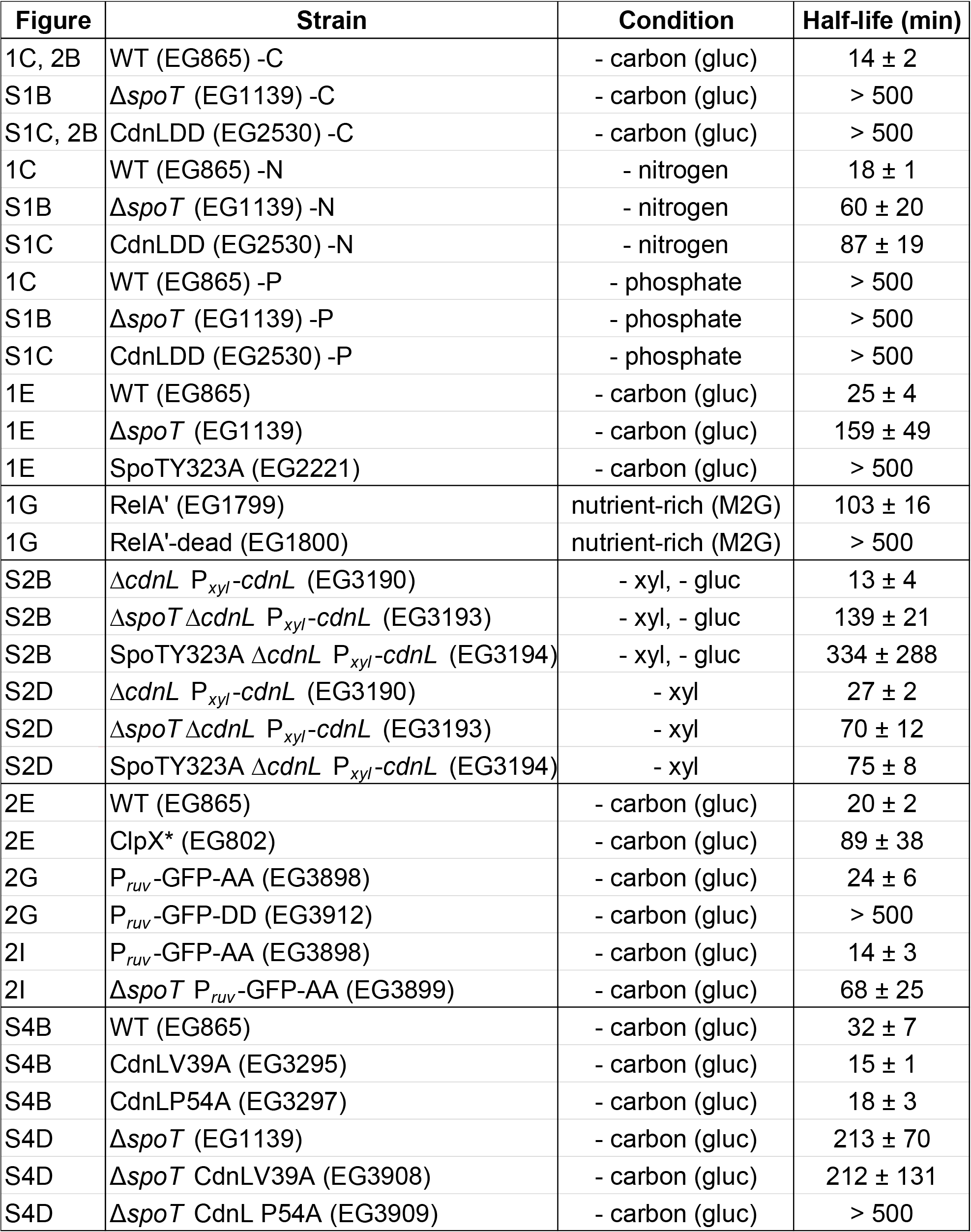
**Supplemental Table S1: Half-life values**

Day-to-day variability can be seen between experiments; however, each experiment is internally controlled for, and comparisons are made within experiments. Error indicates +/- 1 SD of 3 biological replicates

**Supplemental Table S2: Outgrowth and doubling times for Figure 3
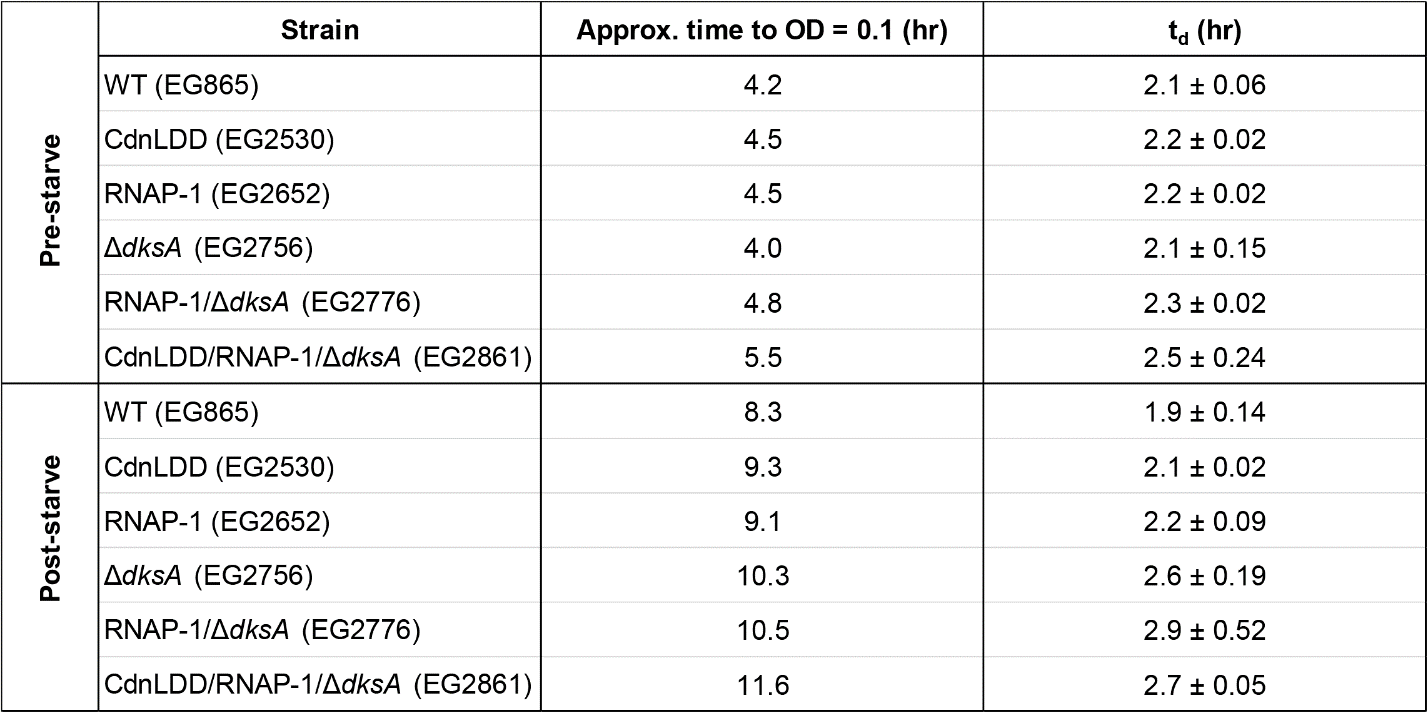
**

Error indicates +/- 1 SD of 3 biological replicates

**Supplemental Table S3: Plasmids and Strains used in this study**Attached as a separate Excel file

**Dataset S1: ChIP-seq data of WT CdnL and CdnLDD in M2G and 60 minutes in M2**

**Dataset S2: RNA-seq comparing 0 to 60 minutes of carbon starvation in WT (EG865)**

Includes all quantified genes, genes > 2-fold differentially regulated (p < 0.05 and FDR < 0.05), and overlap with ChIP-seq peaks

**Dataset S3: RNA-seq comparing 0 to 60 minutes of carbon starvation in CdnLDD (EG82530)**

Includes all quantified genes, genes > 2-fold differentially regulated (p < 0.05 and FDR < 0.05), and overlap with ChIP-seq peaks

**Dataset S4: RNA-seq comparing WT (EG865) to CdnLDD (EG2530) at 0 minutes of starvation**

Includes all quantified genes, genes > 2-fold differentially regulated (p < 0.05 and FDR < 0.05), and DAVID analyses

**Dataset S5: RNA-seq comparing WT (EG865) to CdnLDD (EG2530) at 60 minutes of starvation**

Includes all quantified genes, genes > 2-fold differentially regulated (p < 0.05 and FDR < 0.05), and DAVID analyses

**Dataset S6: RNA-seq data comparing WT (EG865) 60 minutes starved to 24 hours starved**

Includes all quantified genes and genes > 2-fold differentially regulated (p < 0.05 and FDR < 0.05)

**Dataset S7: RNA-seq comparing WT (EG865) to CdnLDD (EG2530) at 24 hours of starvation**

Includes all quantified genes, genes > 2-fold differentially regulated (p < 0.05 and FDR < 0.05), and DAVID analyses

**Dataset S8: RNA-seq comparing WT (EG865) to CdnLDD (EG2530) at 60 minutes of recovery after glucose addition**

Includes all quantified genes, genes > 2-fold differentially regulated (p < 0.05 and FDR < 0.05), and DAVID analyses
